# Supplementary material for: C/EBPβ enhances platinum resistance of ovarian cancer cells by reprogramming H3K79 methylation
Source: Nat Commun. 2018 Apr 30;9:1739. doi: 10.1038/s41467-018-03590-5 (PMC5928165; doi:10.1038/s41467-018-03590-5)
Supplement: Supplementary file 1 — Supplementary Information(PDF 6102 kb) [file 41467_2018_3590_MOESM1_ESM.pdf]

Supplementary Table 1. Characteristics of patients used in high-throughput sequencing study.

No.	Age at diagnosis	FIGO stage	Histologic type	*P53* mutation			Ascites	CA125# (U/ml)
				cDNA	Protein	Mutation type		
1	43	II	HG-SOC	817C>T	R273C	Missense	no	93.8
2	58	II	HG-SOC	524G>A	R175H	Missense	no	140
3	42	III	HG-SOC	844C>T	R282W	Missense	yes	842.7
4	59	III	HG-SOC	743G>A	R248Q	Missense	no	768
5	43	III	HG-SOC	733G>A	G245S	Missense	yes	2720.5
6	48	III	HG-SOC	584T>C	I195T	Missense	yes	3474.4
7	57	III	HG-SOC	681_682insT	D228fs*	Frameshift	yes	6756.1
8	59	III	HG-SOC	524G>A	R175H	Missense	yes	8777
9	60	III	HG-SOC	818G>A	R273H	Missense	yes	5442
10	49	III	HG-SOC	659A>G	Y220C	Missense	no	447
11	42	III	HG-SOC	844C>T	R282W	Missense	yes	125.7
12	65	III	HG-SOC	743G>A	R248Q	Missense	yes	1868.3
13	63	III	HG-SOC	527G>A	C176Y	Missense	yes	5877
14	39	III	HG-SOC	818G>A	R273H	Missense	no	1955
15	49	III	HG-SOC	445delT	S149fs*	Frameshift	yes	764.3
16	46	IV	HG-SOC	641A>G	H214R	Missense	yes	>10000
17	48	IV	HG-SOC	396G>T	K132N	Missense	yes	1365
18	65	IV	HG-SOC	524G>A	R175H	Missense	no	>10000
19	64	IV	HG-SOC	578A>G	H193R	Missense	no	966.6
20	55	IV	HG-SOC	469G>T	V157F	Missense	yes	>10000

Abbreviation: FIGO, International Federation of Gynecology and Obstetrics; HG-SOC, High-grade serous ovarian cancer; fs*, Frameshift.

#CA125: the preoperative CA125 value.

Supplementary Table 2. Characteristics of patients with serous ovarian cancer.

Characteristics	Total Patients (N = 245)		C/EBPβ-low (N = 175)		C/EBPβ-high (N = 70)		P-value
	No.	%	No.	%	No.	%	
Age at diagnosis							0.671
Mean (years)	51.7		51.8		51.3		
Range (years)	25-80						
FIGO stage							0.624
II	15	6.1	12	6.9	3	4.3	
III	194	79.2	139	79.4	55	78.6	
IV	36	14.7	24	13.7	12	17.1	
Ascites							0.168
Yes	162	66.1	118	72.0	44	62.9	
No	72	29.4	46	28.0	26	37.1	
Unknown	11	4.5					
Residual disease							0.348
R0	106	43.3	79	45.1	27	38.6	
R1	139	56.7	96	54.9	43	61.4	

Abbreviation: FIGO, International Federation of Gynecology and Obstetrics.

R0: no gross residual; R1: any residual.

Supplementary Table 3. Univariate analysis of the prognosis in 245 patients with HG-SOC using the Hazards Model (Cox analysis).

Factor	OS			PFS		
	Relative Risk	95% CI	*P*	Relative Risk	95% CI	*P*
C/EBPβ expression (low *v* high)	2.370	1.508-3.725	0.0002	3.958	2.795-5.606	9.36×10^{-15}
Residual disease (R0 *v* R1)	3.614	2.306-5.664	2.09×10^{-8}	2.147	1.525-3.022	1.20×10^{-5}
Age at diagnosis (≤ 55 *v* > 55 years)	1.571	1.008-2.450	0.046	0.983	0.673-1.436	0.929
FIGO stage (II *v* III, IV)	4.138	1.016-16.855	0.048	1.490	0.729-3.045	0.274
Ascites (No *v* Yes)	0.905	0.567-1.443	0.905	0.821	0.570-1.182	0.289

Abbreviation: FIGO, International Federation of Gynecology and Obstetrics.

R0: no gross residual; R1: any residual.

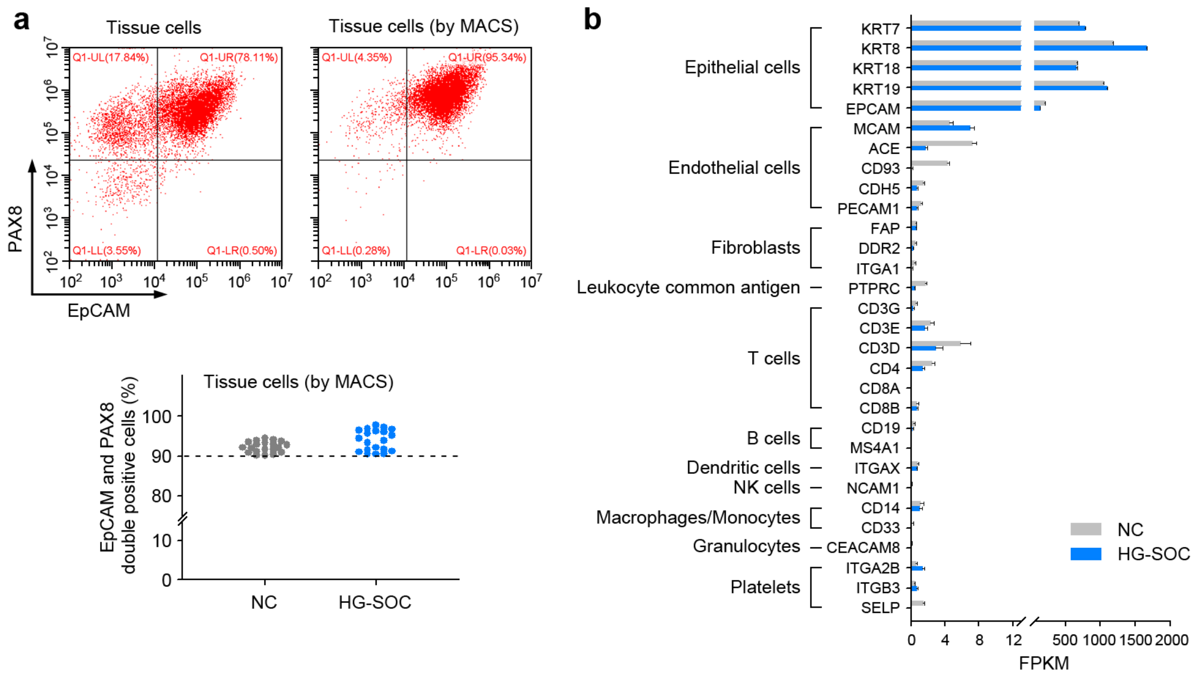

Supplementary Figure 1. Quality control of magnetically isolated tissue cells. a, The percentages of EpCAM and PAX8 positive cells before and after magnetic separations were detected by flow cytometric analysis. MACS, magnetic activated cell sorting. **b**, Tissue type–specific expression profiles of the magnetically isolated samples, measured by RNA-seq. FPKM, fragments per kilobase of exon per million fragments mapped. Error bars indicating 95% confidence interval.

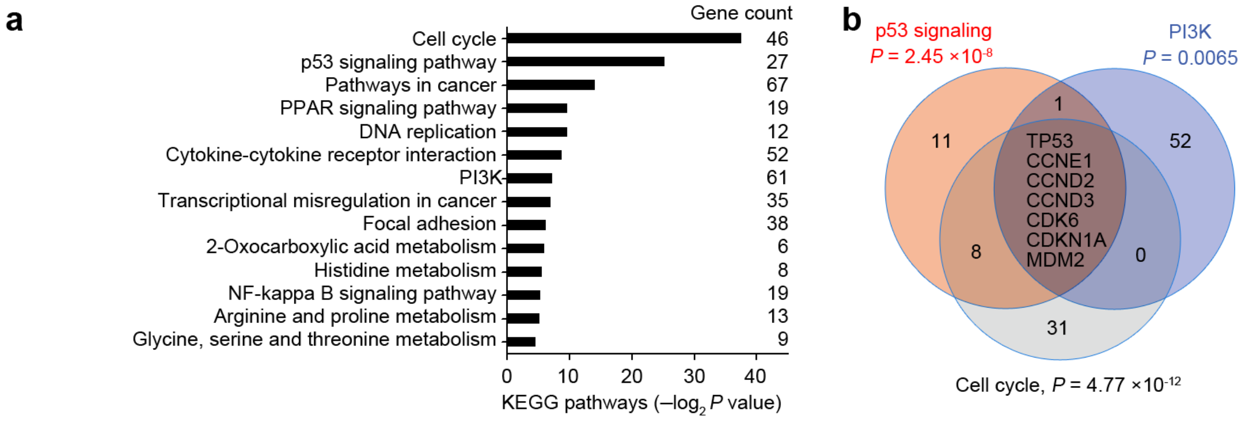

Supplementary Figure 2. TP53 associated signaling pathways were enriched in HG-SOC. **a**, KEGG pathway analysis of the differentially expressed genes in HG-SOC compared to normal fallopian tube. **b**, Venn diagrams showing the overlap of genes in p53 signaling, cell cycle and PI3K pathway.

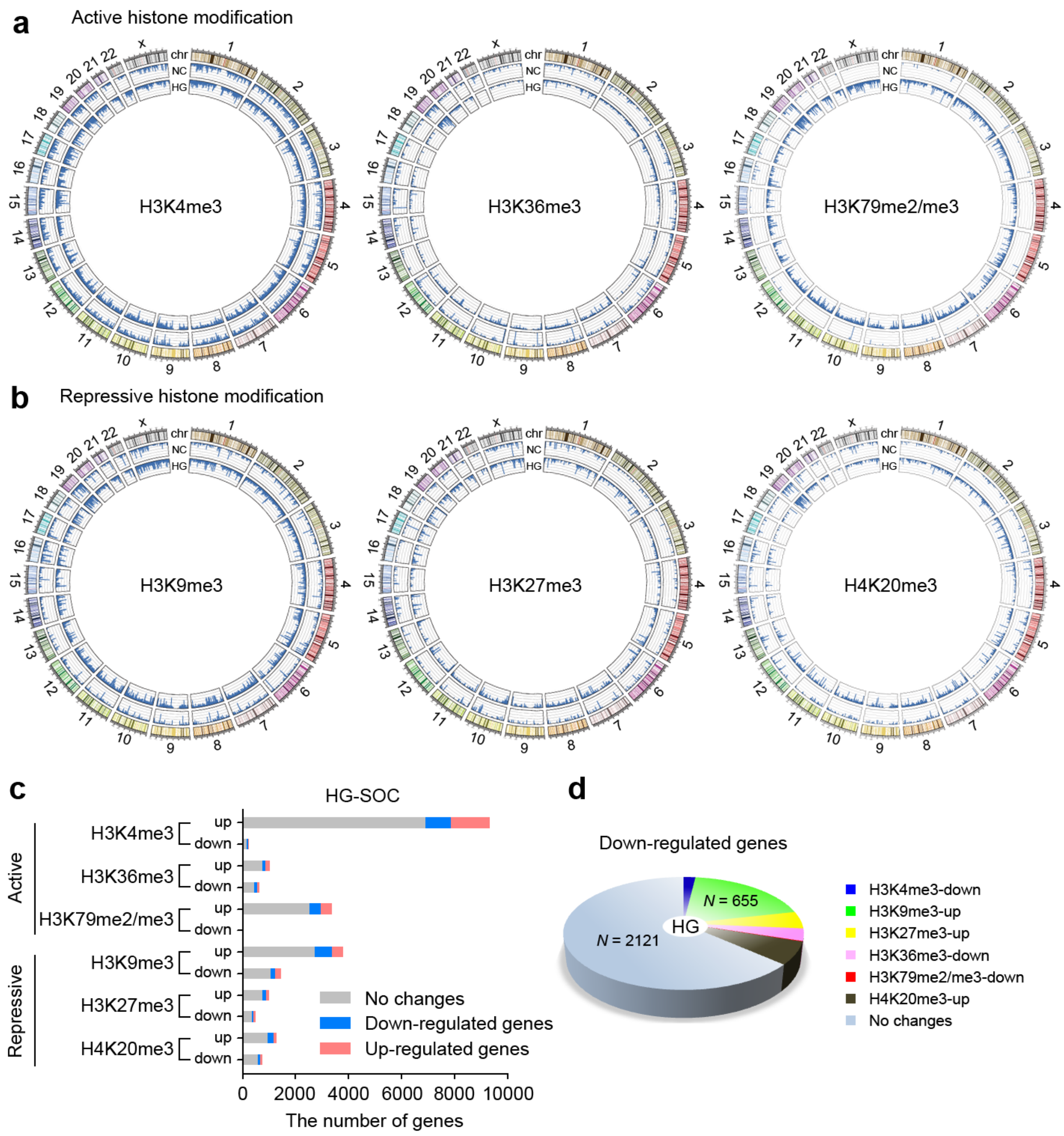

Supplementary Figure 3. Histone methylation profiles and transcript profiles of human serous ovarian cancer and normal fallopian tube samples. Magnetic separation of epithelial cells and preparation of pooled samples from 20 HG-SOC and 20 normal fallopian tubes were performed. **a**, **b**, Global profiles for ChIP-seq signals of the active (**a**) and repressive (**b**) histone methylation sites in purified and pooled samples. The histogram axis scale represents read density per million sequenced reads and the outer DNA numbering is given in millions of bases. chr, chromatin; HG, HG-SOC; NC, normal control (normal fallopian tubes). **c**, The differentially methylated chromatins and differentially expressed genes in HG-SOC samples were identified using ChIP-seq ($P < 10^{-4}$, fold change > 2) and RNA-seq ($P < 0.05$), respectively. The numbers of genes in each group are shown. d, Pie diagrams showing the numbers of downregulated genes coupled with corresponding changes in the indicated histone methylation sites (HG-SOC versus NC group).

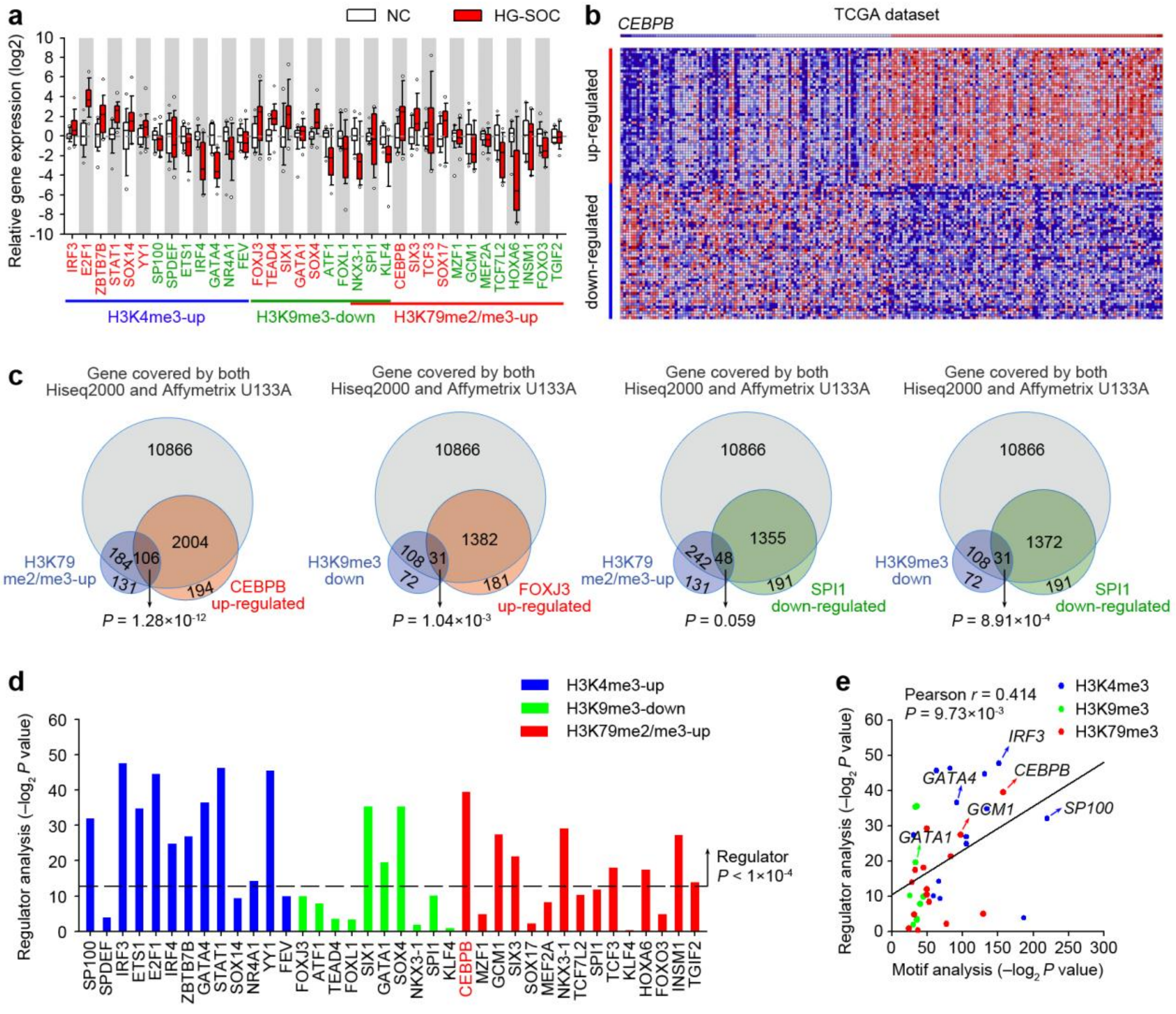

Supplementary Figure 4. Gene expression regulation analysis of the transcription factors. **a**, The relative mRNA levels of the indicated transcription factors in the magnetically separated HG-SOC (n = 20) and normal fallopian tube (NC, n = 20) samples were detected by RT-qPCR. Boxes represent upper quartile, median and lower quartile; whiskers represent the 5–95th percentiles of the data. The mean values of relative gene expression in normal fallopian tube group were normalized to 1. **b**, Predicting the downstream genes of transcription factors using TCGA dataset. For each gene, the lower 200 and the higher 200 of 489 HG-SOC samples in TCGA dataset were defined as low- and high-expression group, respectively. Differential gene expression was analysis by Significance Analysis of Microarrays (SAM version 4.01). Representative heat map is shown. Columns represent different samples and are plotted according to *CEBPB* expression increased from left to right. Blue represent low expression; Red represent high expression. **c**, Regulator analysis. Venn diagrams showing the overlap of indicated gene groups, in the gene set covered by both U133A microarray and next generation sequencing (chi-squared tests). The examples of up- and down-regulated transcription factors are also shown. **d**, Summarizing the *P* values of each regulator analysis. **e**, The correlation of regulator analysis and motif analysis.

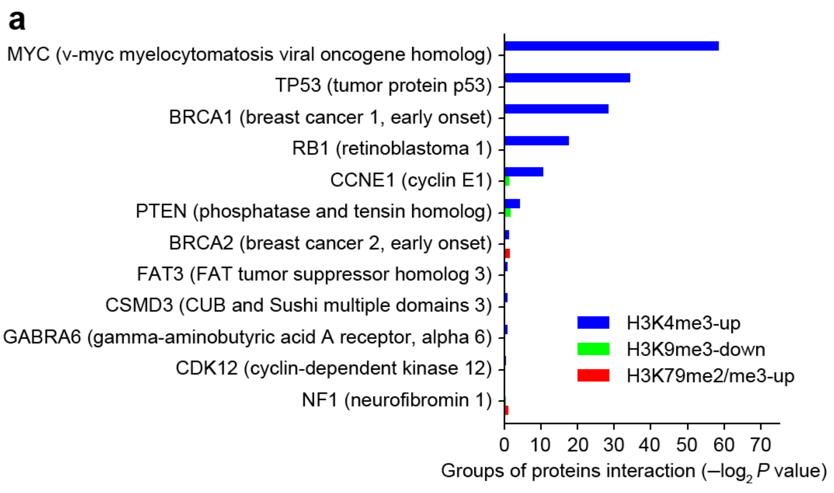

Supplementary Figure 5. TP53 associated signaling pathways were enriched in HG-SOC. **a**, Analysis of protein-protein interaction. Analyze groups of epigenetically altered genes that interacting with the same protein. **b**, Venn diagrams showing the overlap of genes interact with MYC, TP53, BRCA1, RB1 and CCNE1.

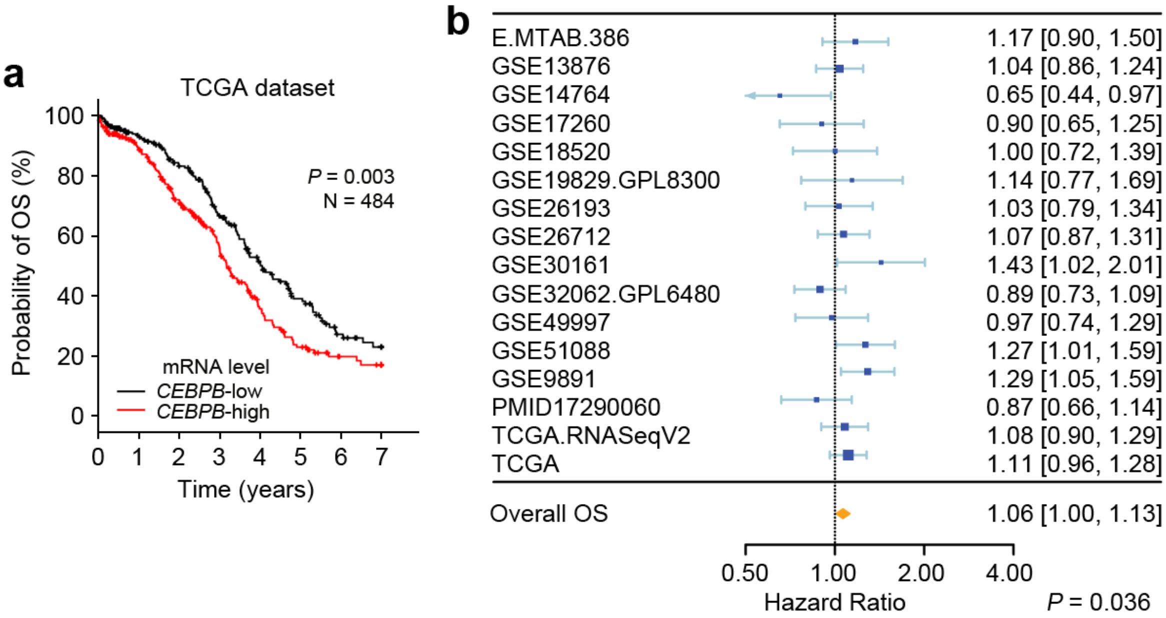

Supplementary Figure 6. Prognosis analysis of the regulators for histone methylation. a, Analysis of OS for each transcription factor using TCGA dataset. Samples were divided into two groups based on the median value of mRNA levels. Representative survival curve is shown. OS, overall survival. **b**, Forest plot of the expression of *CEBPB* as a univariate predictor of OS in patients with ovarian cancer. Gene expression meta-analysis was performed using the 'curatedOvarianData_1.3.5' Bioconductor package. The size of the blue boxes for each outcome represents the weight given to that outcome; horizontal lines represent 95% CIs; diamonds represent summary estimates with corresponding 95% CIs. OS, overall survival.

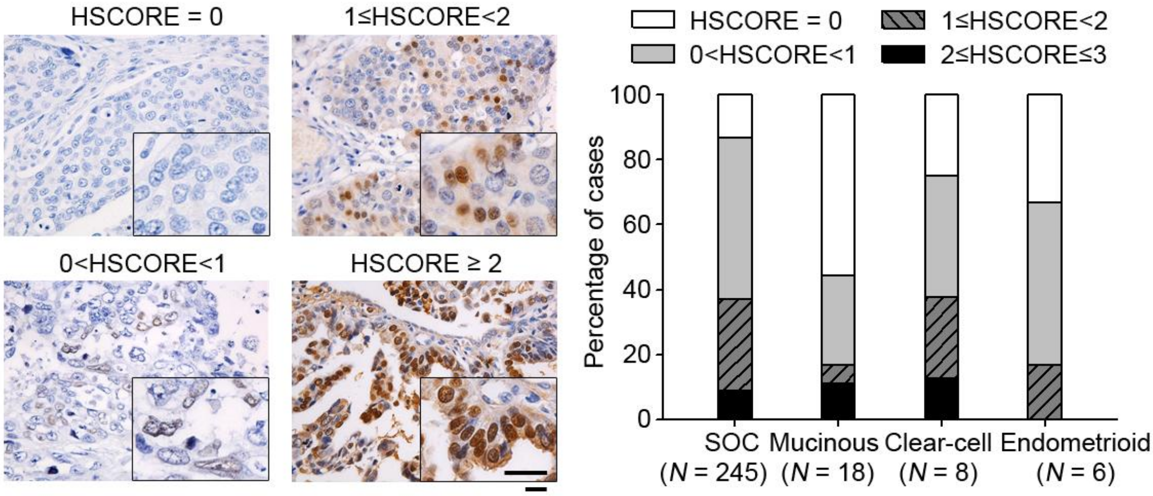

Supplementary Figure 7. IHC analysis of C/EBPβ protein in 4 histologic types of human epithelial ovarian cancer. Representative images of C/EBPβ staining are shown at ×400 magnification (left); insets are enlarged local images. Bar, 25 μm. The IHC scores (HSCORE) of C/EBPβ are also shown (right).

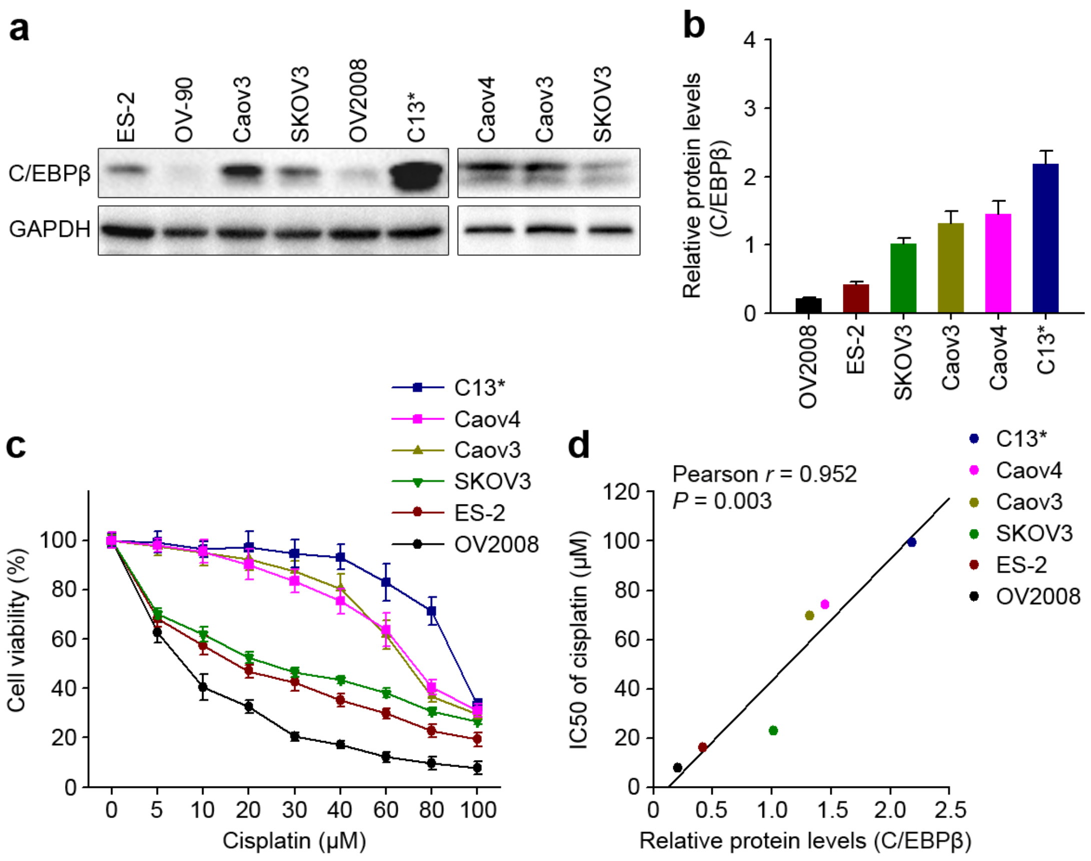

Supplementary Figure 8. Relationship between C/EBPβ protein levels and cisplatin resistance in ovarian cancer lines. a, C/EBPβ expression in the indicated ovarian cancer cell lines was detected by western blotting. **b**, C/EBPβ expression in the indicated ovarian cancer cell lines was detected by western blotting. GAPDH was used as a loading control. 3 independent experiments were performed. **c**, Cisplatin resistance of the six cell lines was determined at 36 h following a series of cisplatin concentrations using the CCK8 assay. The assay was performed using 5 replicates. The half-maximal inhibitor concentration (IC50) of cisplatin for each cell line was determined by nonlinear regression analysis. **d**, Correlation analysis of the relative C/EBPβ protein level and the IC50 of cisplatin was performed, indicating that higher levels of C/EBPβ were associated with higher intrinsic resistance to cisplatin. Correlation index r and *P* value were calculated using Pearson's correlation test. Uncropped images of blots are shown in Supplementary Fig. 25.

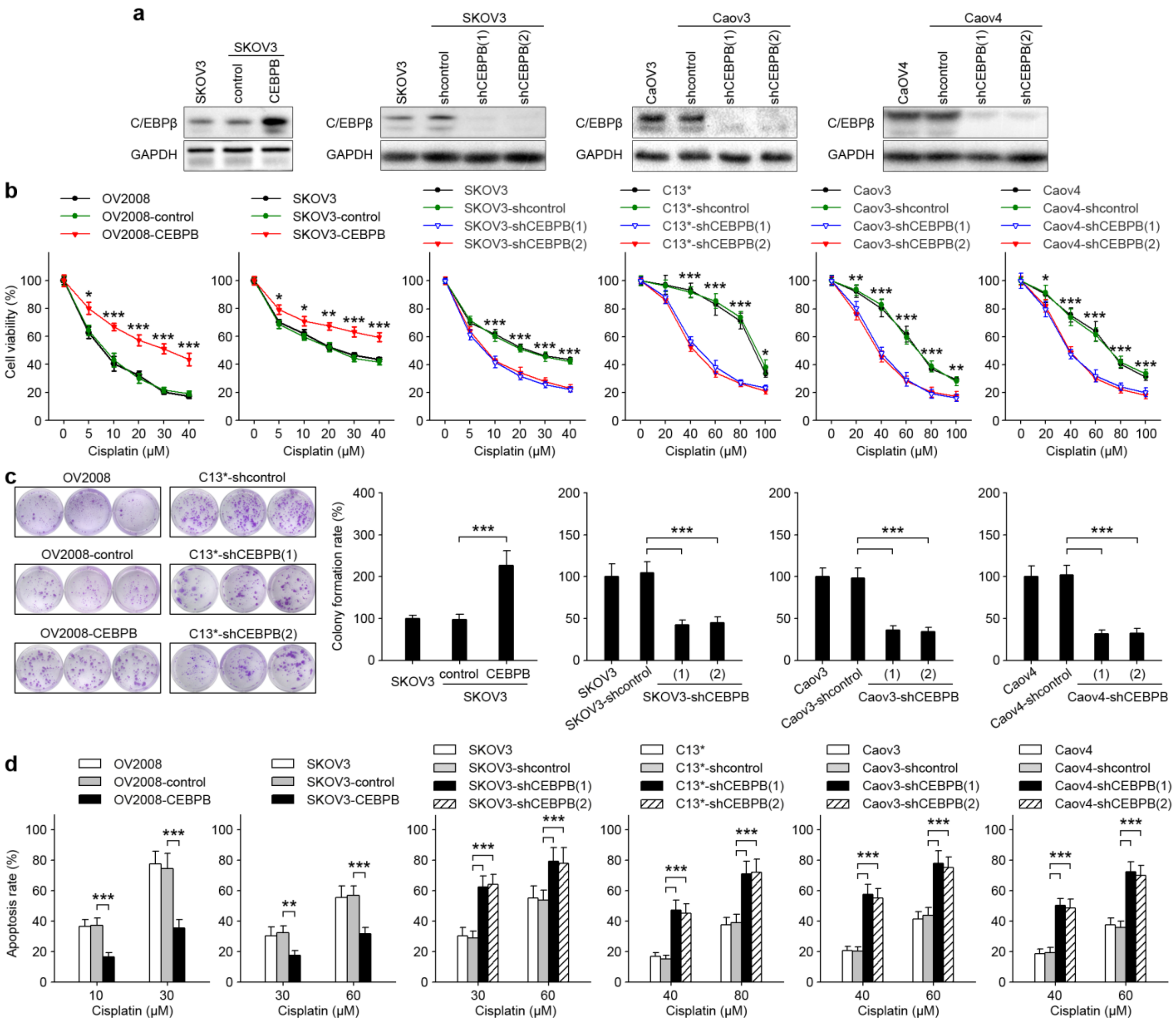

Supplementary Figure 9. C/EBPβ promotes cisplatin resistance of ovarian cancer cells *in vitro*. a, C/EBPβ expression in the indicated cells was detected by western blotting. Two nonoverlapping shRNAs were used to knockdown the expression of C/EBPβ. **b**–**d**, Assay of cisplatin resistance *in vitro*. Cisplatin resistance of the indicated cell lines was determined by the CCK8 assay (at 36 h following a series of cisplatin concentrations) (**b**), standard colony formation assay (after exposure to 50 μM cisplatin for 12 hours) (**c**) and apoptosis assay (at 24 h following the indicated cisplatin concentrations) (**d**). Uncropped images of blots are shown in Supplementary Fig. 25.

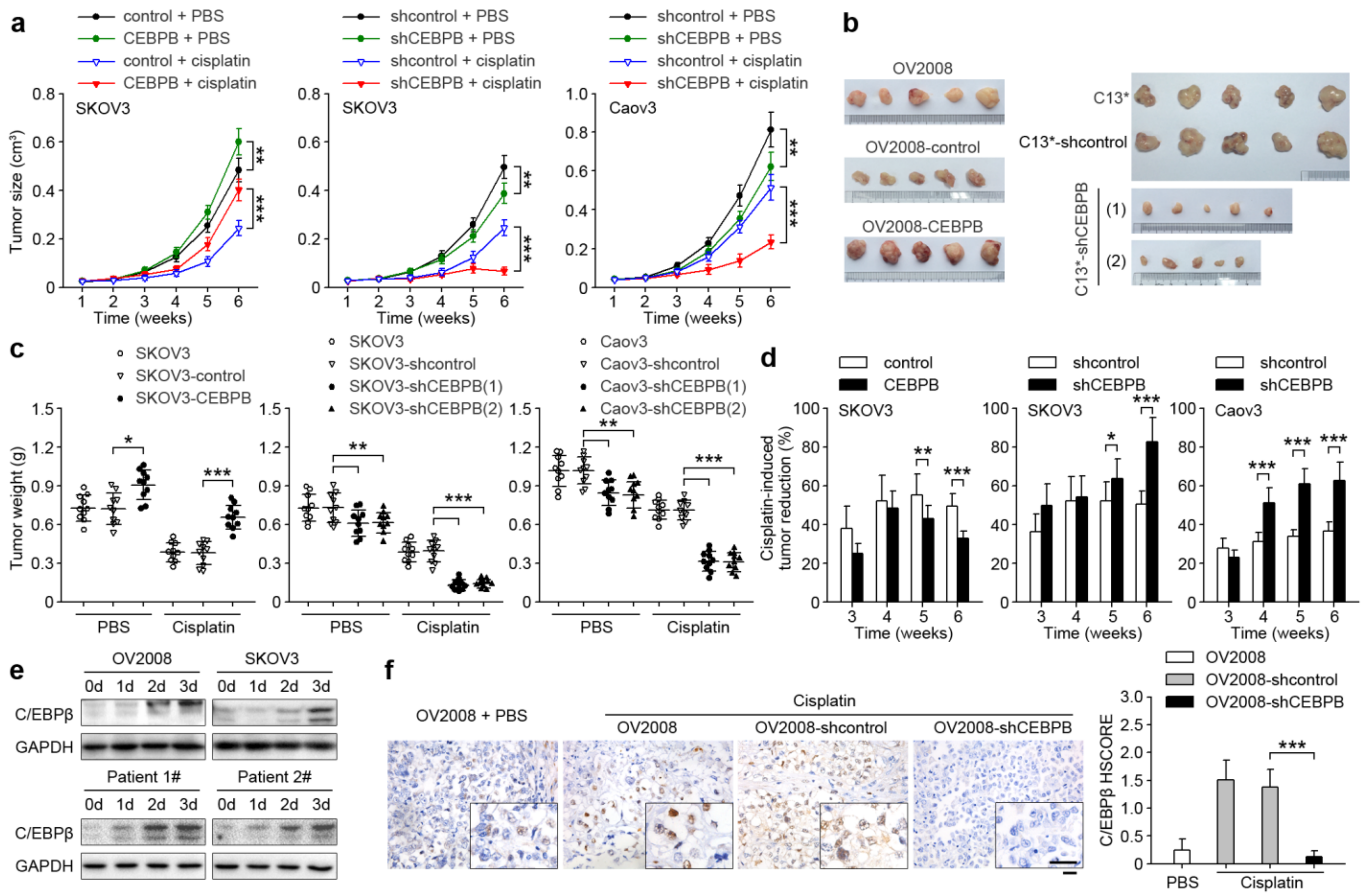

Supplementary Figure 10. C/EBPβ promotes cisplatin resistance of ovarian cancer cells *in vivo*. One week after orthotopical inoculation with the indicated cells, the mice were treated with cisplatin (5 mg/kg) or vehicle (PBS) intraperitoneally every 4 days (n = 10 per group). **a**, The size of tumor was monitored by three-dimensional reconstruction of *in vivo* bioluminescence images. **b**, **c**, Six weeks after tumor inoculation, tumors were excised (**b**) and weighted (**c**). **d**, Cisplatin-induced tumor reduction rate was calculated as described in Methods, using the following formula: $(1 - V_{cisplatin}/V_{PBS}) \times 100\%$. **e**, OV2008 cells, SKOV3 cells, or primary cultures of tumor cells from patients with ovarian cancer were treated with cisplatin (30 μM) for different times, and C/EBPβ expression was then detected by western blotting. **f**, IHC analysis of C/EBPβ protein in xenograft tumor sections collected from mice. Representative images of C/EBPβ staining are shown at ×400 magnification (left); insets are enlarged local images. Bar, 25 μm. Uncropped images of blots are shown in Supplementary Fig. 25.

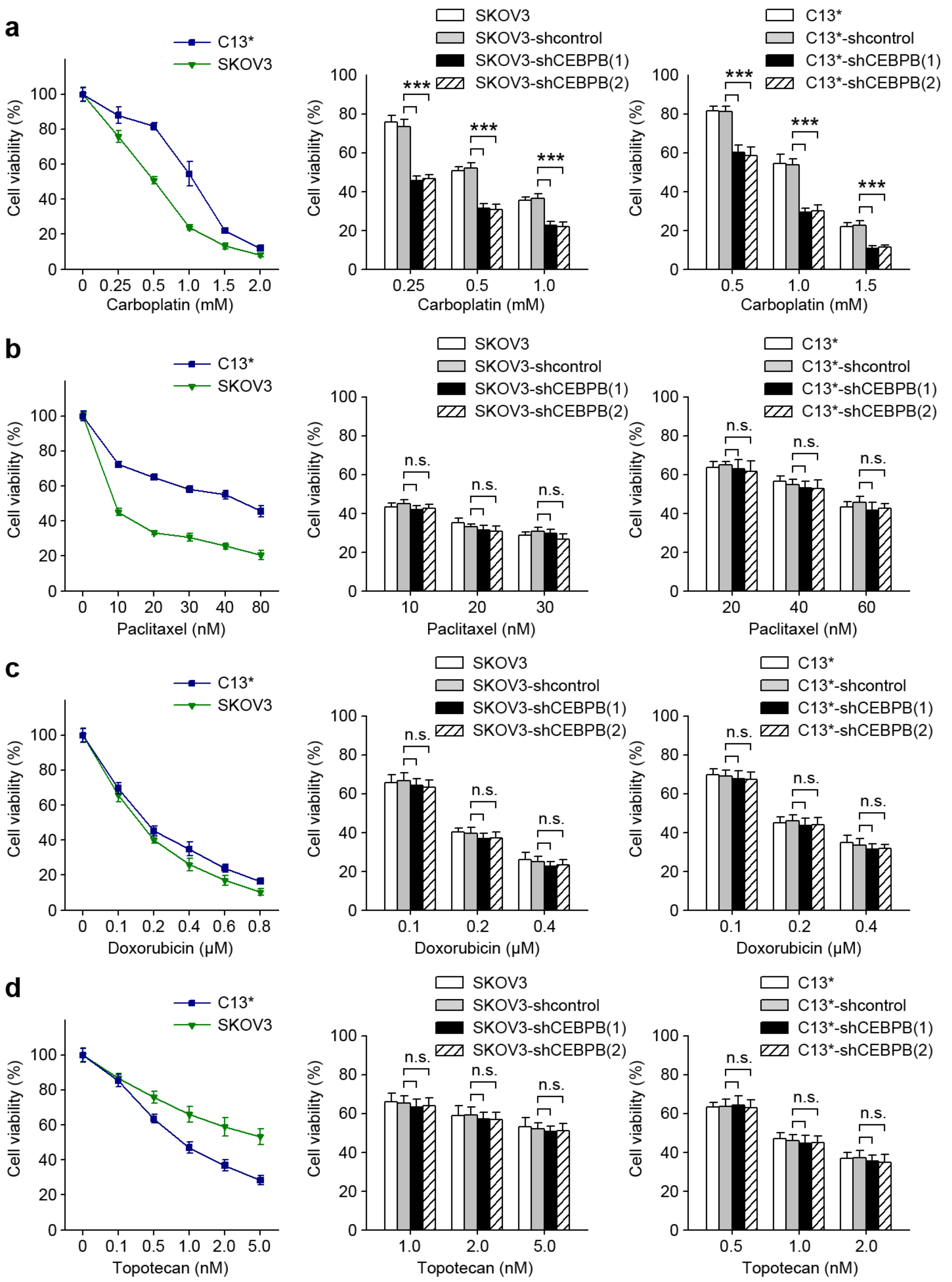

Supplementary Figure 11. Effect of C/EBPβ on the sensitivity of other commonly used chemotherapy drugs in ovarian cancer. The indicated cell lines were treated with a series of concentrations of carboplatin (**a**), paclitaxel (**b**), doxorubicin (**c**) and topotecan (**d**) for 36 h. Then cell viability was determined by the CCK8 assay. The assay was performed using 5 replicates.

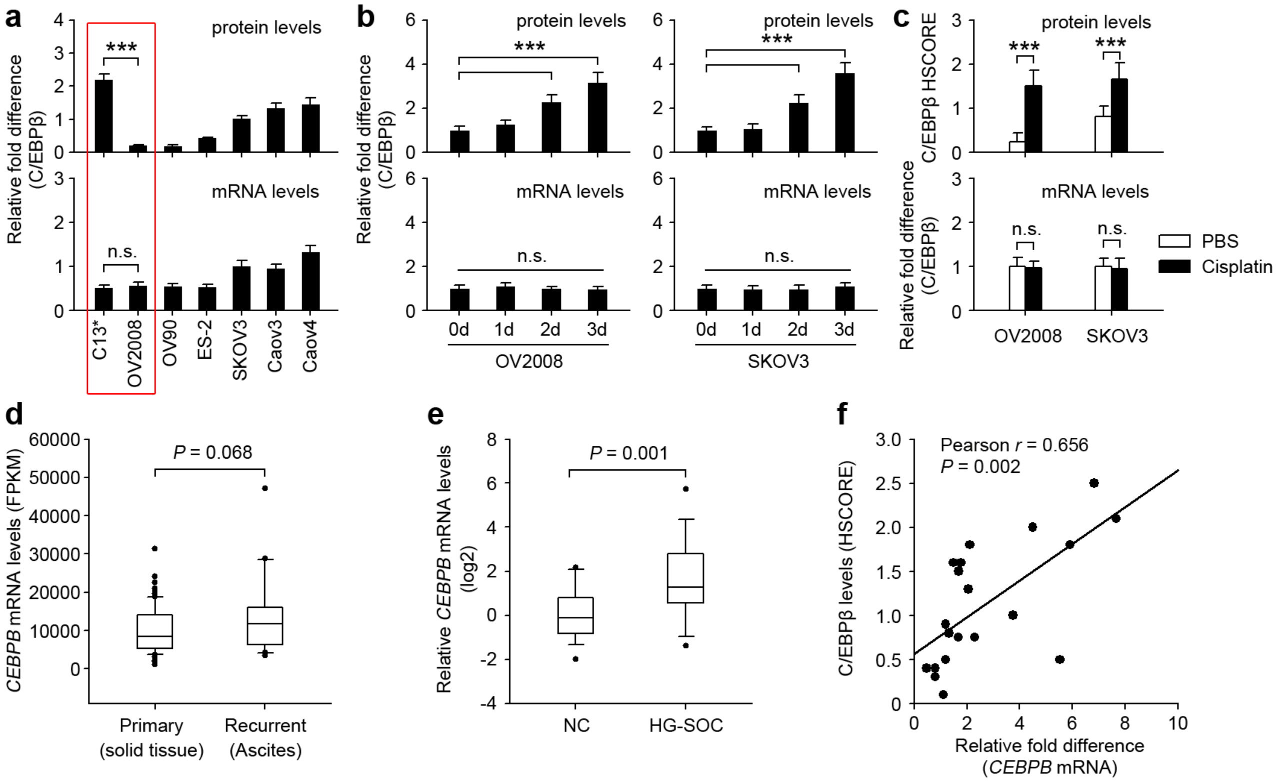

Supplementary Figure 12. Different mechanisms for C/EBPβ upregulation in tumorigenesis and after chemotherapy. a, C/EBPβ protein levels (upper panel) and mRNA levels (lower panel) in the indicated ovarian cancer cell lines were detected by western blotting and RT-qPCR, respectively. **b**, OV2008 cells and SKOV3 cells were treated with cisplatin (30 μM) for different times, and C/EBPβ protein levels (upper panel) and mRNA levels (lower panel) were detected by western blotting and RT-qPCR, respectively. **c,** C/EBPβ protein levels (upper panel) and mRNA levels (lower panel) in xenograft tumor sections collected from mice treated with or without cisplatin were detected by IHC analysis and RT-qPCR, respectively. **d**, Analysis of gene differential expression of *CEBPB* mRNA between primary HG-SOC (solid tissue) and recurrent HG-SOC (ascites specimens), using European Genome-phenome Archive (EGA) controlled data under the accession code EGAD00001000877. FPKM, fragments per kilobase of exon per million fragments mapped, measured by RNA-seq. Boxes represent upper quartile, median and lower quartile; whiskers represent the 5–95th percentiles of the data. **e**, The relative *CEBPB* mRNA levels in the magnetically separated HG-SOC (n = 20) and normal fallopian tube (NC, n = 20) samples were detected by RT-qPCR. These are the same data showed in Supplementary Figure 4a. **f**, The correlation analysis of *CEBPB* mRNA levels (detected by RT-qPCR) and C/EBPβ protein levels (detected by IHC analysis) in HG-SOC specimens (n = 20).

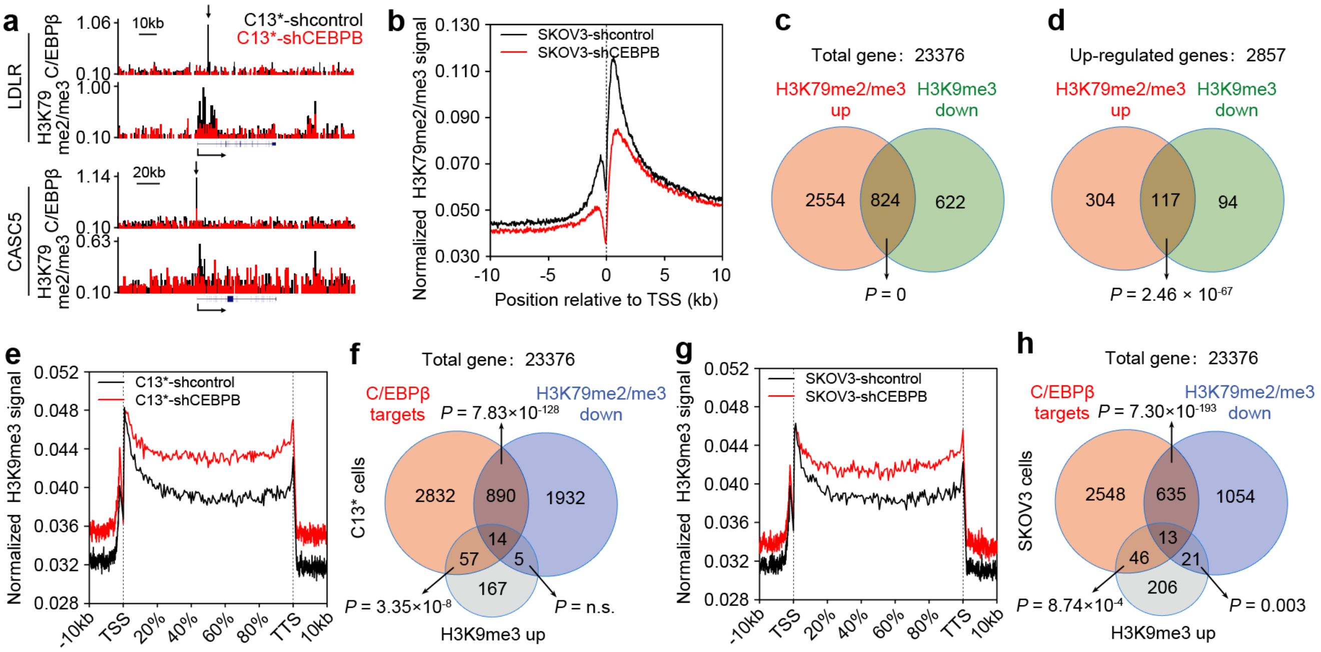

Supplementary Figure 13. C/EBPβ promotes H3K79 methylation. **a**, Profiles for normalized C/EBPβ ChIP-seq signals and H3K79me2/me3 ChIP-seq signals relative to representative C/EBPβ-targeted genes (*LDLR* and *CASC5*). **b**, Meta-analysis of the averaged H3K79me2/me3 ChIP-seq signals of genes in indicated SKOV3 cells. **c** and **d**, Venn diagrams showing the overlap of H3K79me2/me3-up and H3K9me3-down genes (HG-SOC versus normal fallopian tubes, chi-squared tests) in the total gene set (c) and in the upregulated gene set (d). **e**, Meta-analysis of the averaged H3K9me3 ChIP-seq signals of genes along the transcription unit. The gene body length is aligned by percentage from the transcriptional start site (TSS) to transcription termination site (TTS). 10 kilobases upstream of TSS and 10 kb downstream of TTS are also included. **f**, Venn diagrams showing the overlap of C/EBPβ-targeted genes (identified by C/EBPβ ChIP-seq in C13*-shcontrol cells), H3K79me2/me3 decreased genes (in C13*-shCEBPB compared with C13*-shcontrol cells identified by H3K79me2/me3 ChIP-seq), and H3K9me3 increased genes (in C13*-shCEBPB compared with C13*-shcontrol cells identified by H3K9me3 ChIP-seq) (chi-squared tests). **g**, Meta-analysis, as in e, of the averaged H3K9me3 ChIP-seq signals of genes in indicated SKOV3 cells. **h**, Venn diagrams, as in f, showing the overlap of C/EBPβ-targeted genes, H3K79me2/me3 decreased genes and H3K9me3 increased genes in C/EBPβ knockdown SKOV3 cells (chi-squared tests).

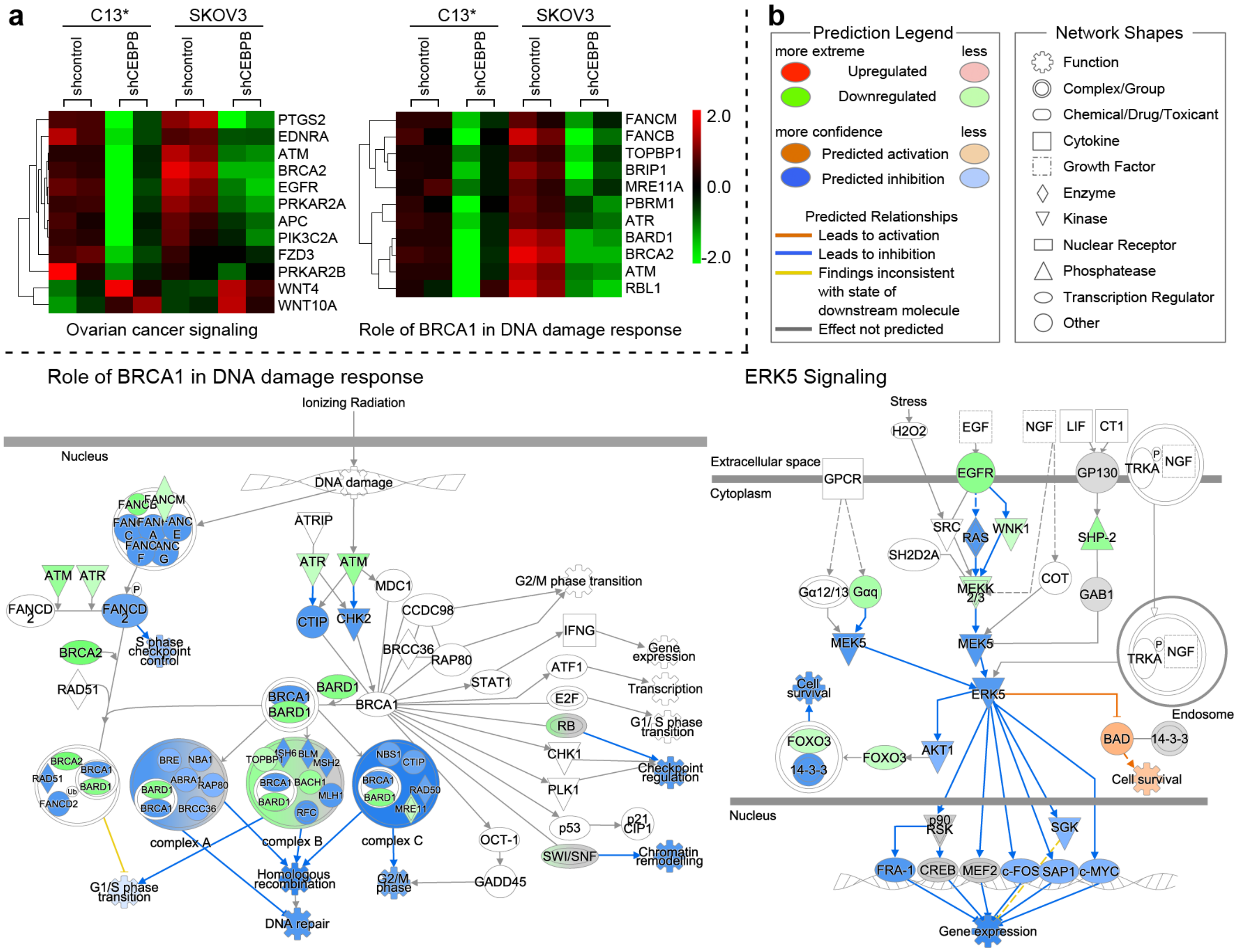

Supplementary Figure 14. Ingenuity pathway analysis (IPA) of the genes that differentially expressed upon C/EBPβ depletion. a, Heatmap of the genes in the two representative pathways identified by IPA. Columns represent individual cell samples, rows correspond to different genes with the expression value z-scores of FPKM (fragments per kilobase of exon per million fragments mapped) as shown in the color bar. **b**, Graphic of the two representative pathways identified by IPA. Down-regulated genes are in green; up-regulated genes are in red. Blue represent suppressed state of the function; orange represent activated state of the function. Lines correspond to physical interactions and arrows to functional relationships between proteins. Solid lines and arrows imply direct relationships and dotted lines and arrows imply indirect relationships. Functional relationships include post-translational modifications, transcription regulation, proteolysis or co-expression. Flat arrowheads indicate inhibition.

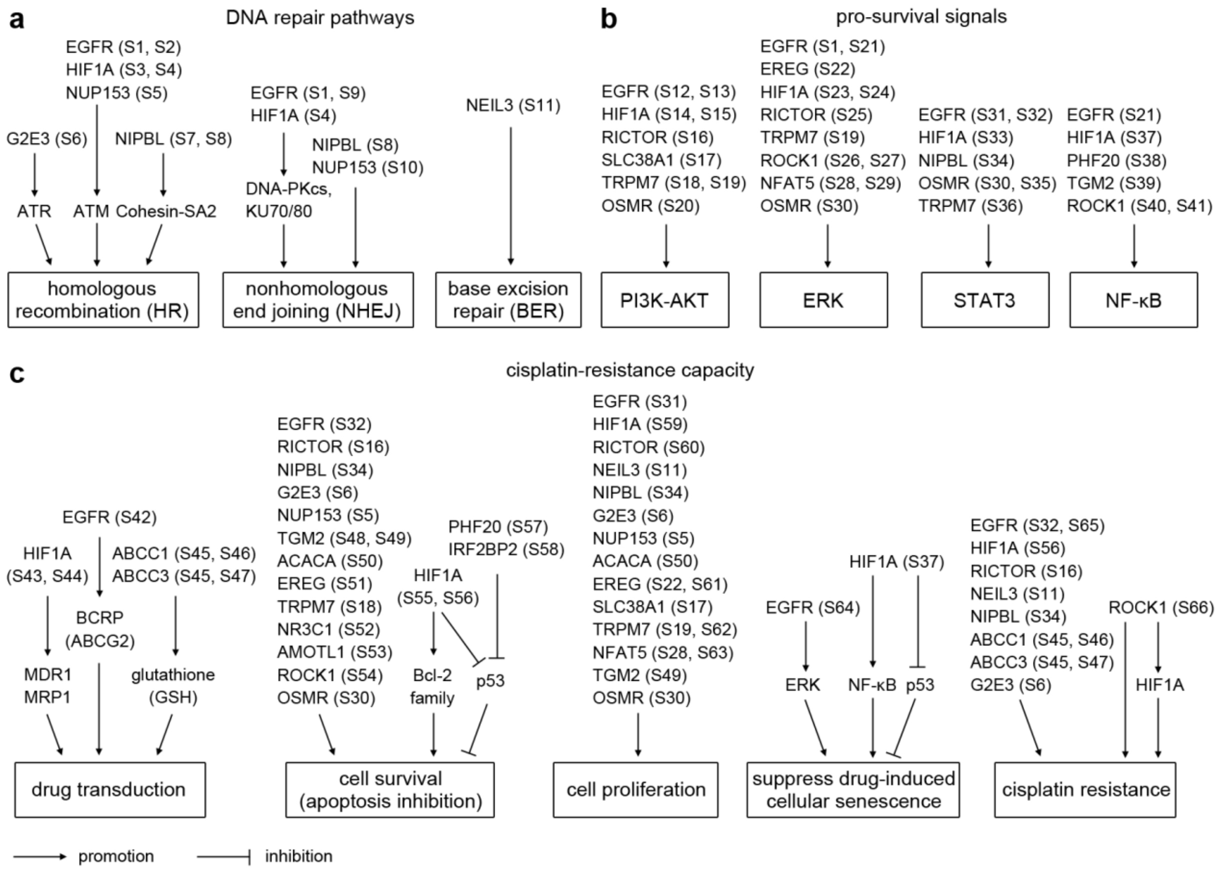

Supplementary Figure 15. The effects of C/EBPβ-targeted genes on signaling pathways and cisplatin-resistance capacity of tumor cells reported in Supplementary References S1 to S66. a, The effect of C/EBPβ-targeted genes on the activation of DNA repair pathways. **b**, The effect of C/EBPβ-targeted genes on the activation of pro-survival signals. **c**, The effect of C/EBPβ-targeted genes on the cisplatin-resistance capacity of tumor cells.

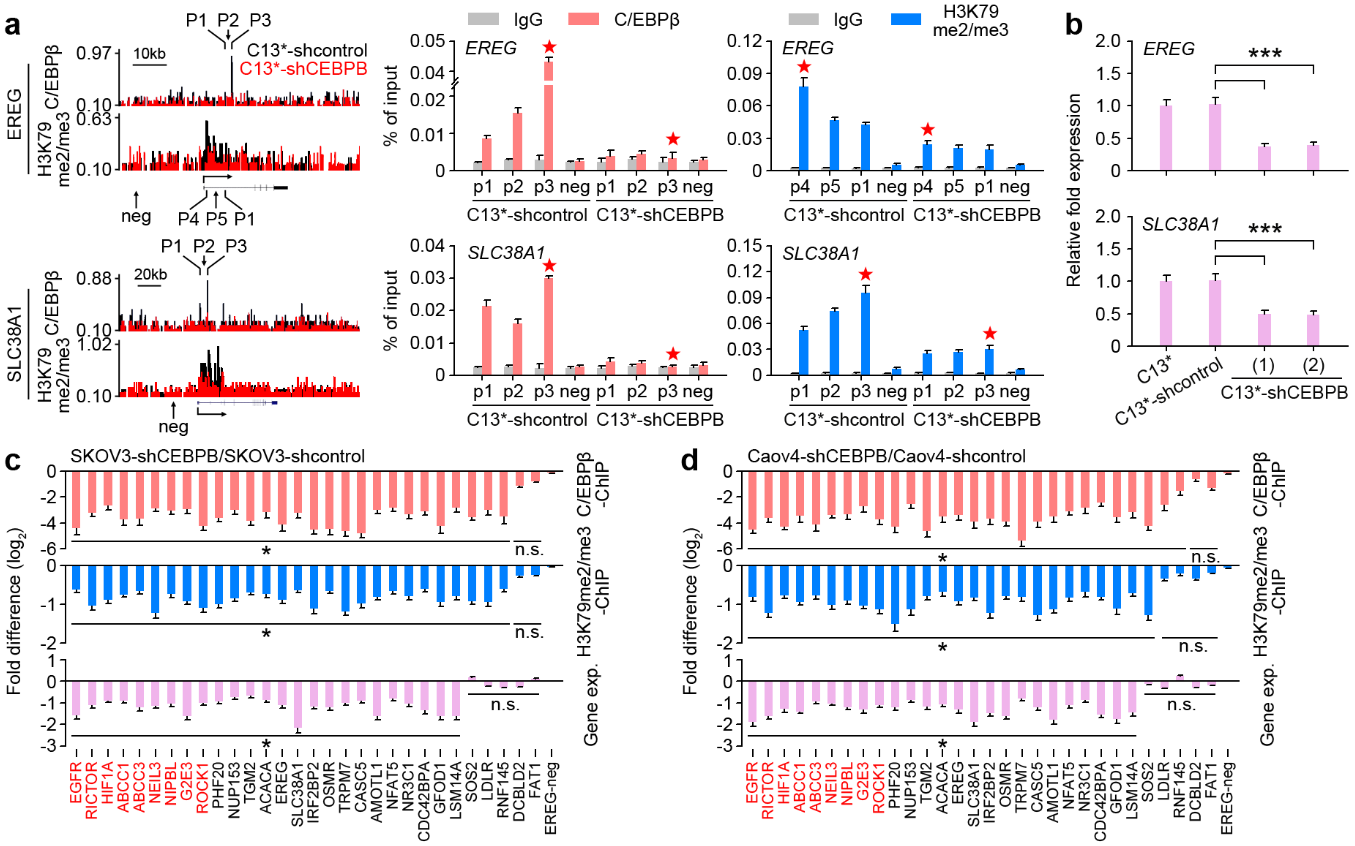

Supplementary Figure 16. C/EBPβ promotes H3K79 methylation and promotes gene expression. a, Representative results of C/EBPβ ChIP-qPCR (pink) and H3K79me2/me3 ChIP-qPCR (blue). ChIP was performed to detect C/EBPβ presence or H3K79me2/me3 presence on the indicated genes in C13* cells. Protein-DNA complexes were precipitated with a C/EBPβ-specific antibody or H3K79me2/me3-specific antibody as well as control IgG antibody, after which qPCR was performed with primers that flank C/EBPβ binding sites or H3K79me2/me3 sites according to the ChIP-seq results. One upstream region with no ChIP-seq signal was used as negative control. One representative result is shown in Figure 4H, using the primers indicated by red pentagrams. **b**, Representative results of relative gene expression analysis using RT-qPCR in C13* cells. **c, d**, Analysis of C/EBPβ ChIP-qPCR (pink) and H3K79me2/me3 ChIP-qPCR (blue), and relative gene expression analysis using RT-qPCR (purple) in SKOV3 cells (**c**) and Caov4 cells (**d**). Gene names in red indicate documented cisplatin-resistance genes in ovarian cancer. *, $P < 0.05$; ***, $P < 0.001$. n.s. indicates “not significant”.

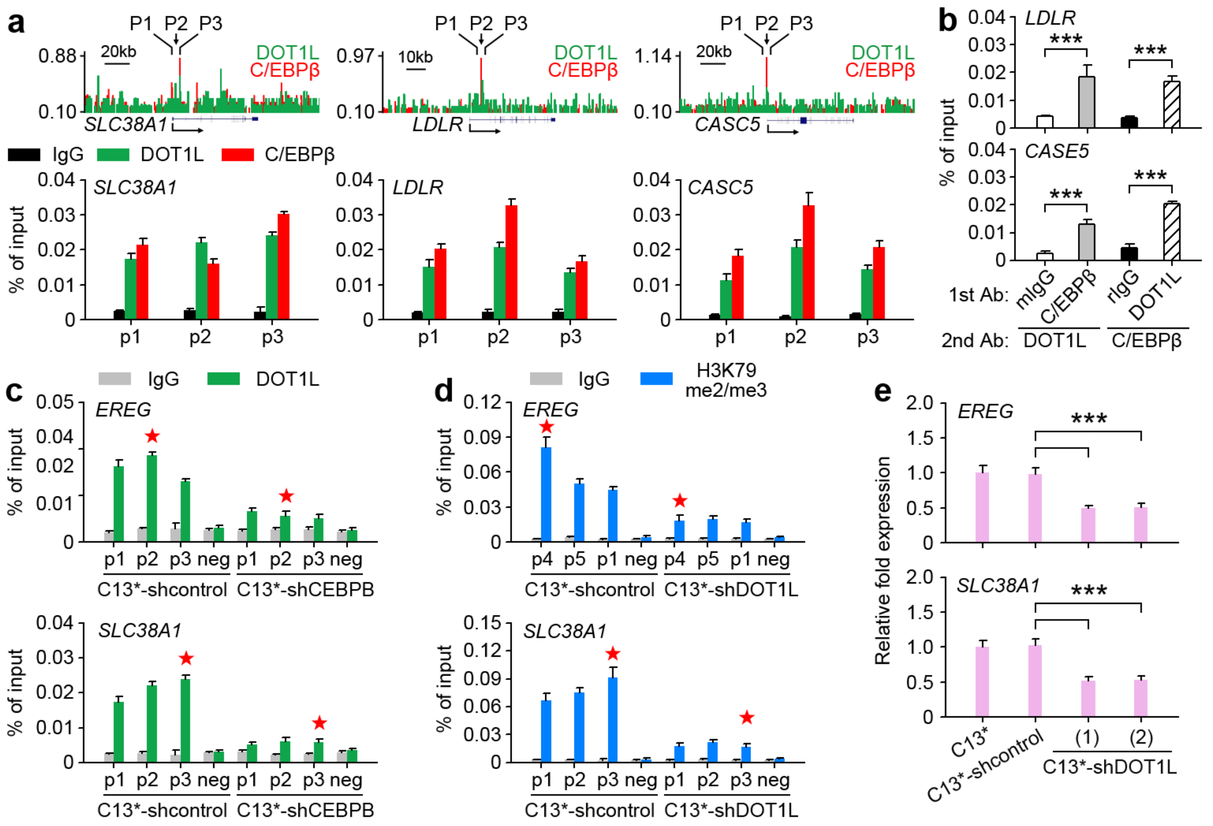

Supplementary Figure 17. C/EBPβ cooperates with DOT1L to regulate gene expression. **a**, Representative results of C/EBPβ ChIP-qPCR (red) and DOT1L ChIP-qPCR (green). Primers were picked flank the binding sites of both C/EBPβ and DOT1L according to the ChIP-seq results (upper panel). **b**, ChIP-reChIP experiments with anti-C/EBPβ and anti-DOT1L antibodies. Mouse IgG (mIgG) and rabbit IgG (rIgG) were used as negative controls. **c**, **d**, ChIP was performed to detect DOT1L presence or H3K79me2/me3 presence on the indicated genes in C13* cells. Protein-DNA complexes were precipitated with a DOT1L-specific antibody or H3K79me2/me3-specific antibody as well as control IgG antibody, after which qPCR was performed with primers that flank DOT1L binding sites or H3K79me2/me3 sites according to the ChIP-seq results. Representative results of DOT1L ChIP-qPCR in C/EBPβ knockdown C13* cells (**c**) and H3K79me2/me3 ChIP-qPCR in DOT1L knockdown C13* cells (**d**). One upstream region with no ChIP-seq signal was used as negative control. One representative result is shown in Figure 5H, using the primers indicated by red pentagrams. **e**, Representative results of relative gene expression analysis using RT-qPCR in DOT1L knockdown C13* cells. ***, $P < 0.001$.

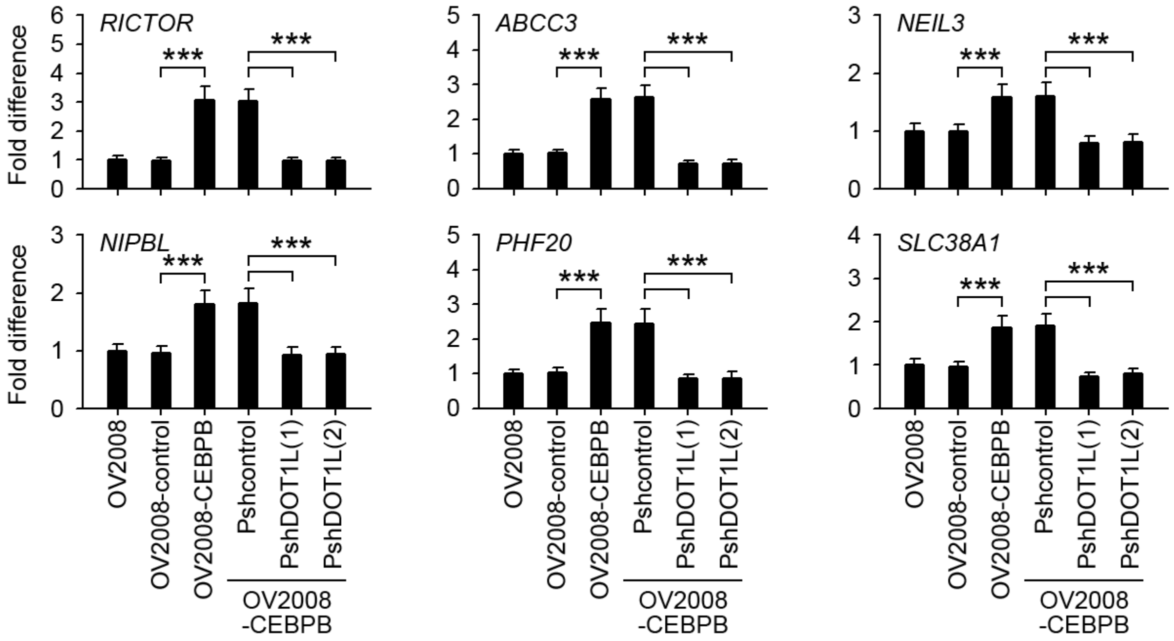

Supplementary Figure 18. DOT1L is necessary for C/EBPβ to promote gene expression in OV2008 cells. The mRNA levels of C/EBPβ-DOT1L co-targeted genes in indicated OV2008 cells were detected by RT-qPCR. ***, $P < 0.001$.

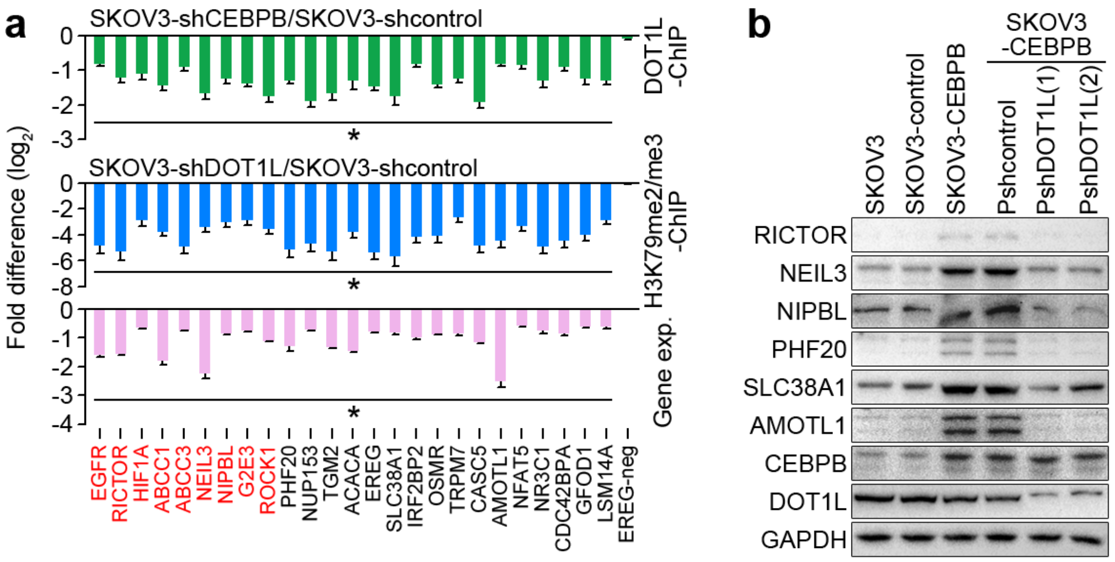

Supplementary Figure 19. DOT1L is necessary for C/EBPβ to promote gene expression in SKOV3 cells. a, Analysis of DOT1L ChIP-qPCR (green) and H3K79me2/me3 ChIP-qPCR (blue), and relative gene expression analysis using RT-qPCR (purple) in SKOV3 cells. Gene names in red indicate documented cisplatin-resistance genes in ovarian cancer. **b**, The protein levels of C/EBPβ-DOT1L co-targeted genes in indicated SKOV3 cells were detected by western blotting. Uncropped images of blots are shown in Supplementary Fig. 26. *, $P < 0.05$.

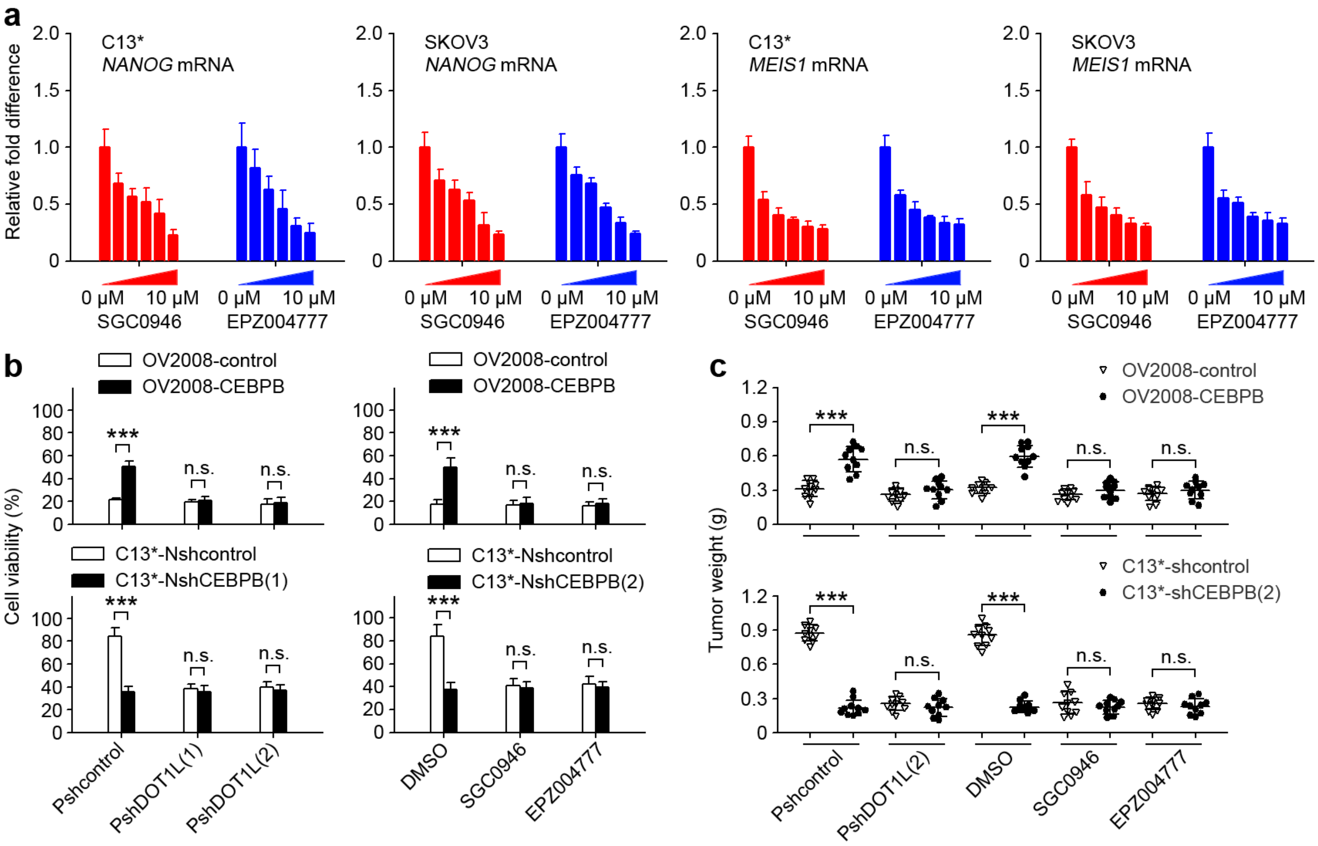

Supplementary Figure 20. The promotional effect of C/EBPβ on cisplatin resistance of tumor cells is mediated by DOT1L. a, Cells were pre-cultured with a series of SGC0946 or EPZ004777 concentrations for up to 12 days. Relative expression levels of DOT1L-targeted genes (*NANOG* and *MEIS1*) were analyzed using RT-qPCR. **b**, cells were pre-cultured in the presence of SGC0946 (5 μM) or EPZ004777 (5 μM) for up to 12 days. Cell viability of the indicated cell lines was determined by the CCK8 assay (at 36 h following 50 μM cisplatin treatment). **c**, Tumor weights. One week after orthotopic inoculation with the indicated cells, the mice were treated with 5 mg/kg cisplatin intraperitoneally every 4 days; for DOT1L inhibitors treatment, 4 mg/kg SGC0946 or EPZ004777 were injected intraperitoneally every 4 days. 6 weeks after tumor inoculation, tumors were excised and weighted (n = 10 per group). ***, $P < 0.001$. n.s. indicates “not significant”.

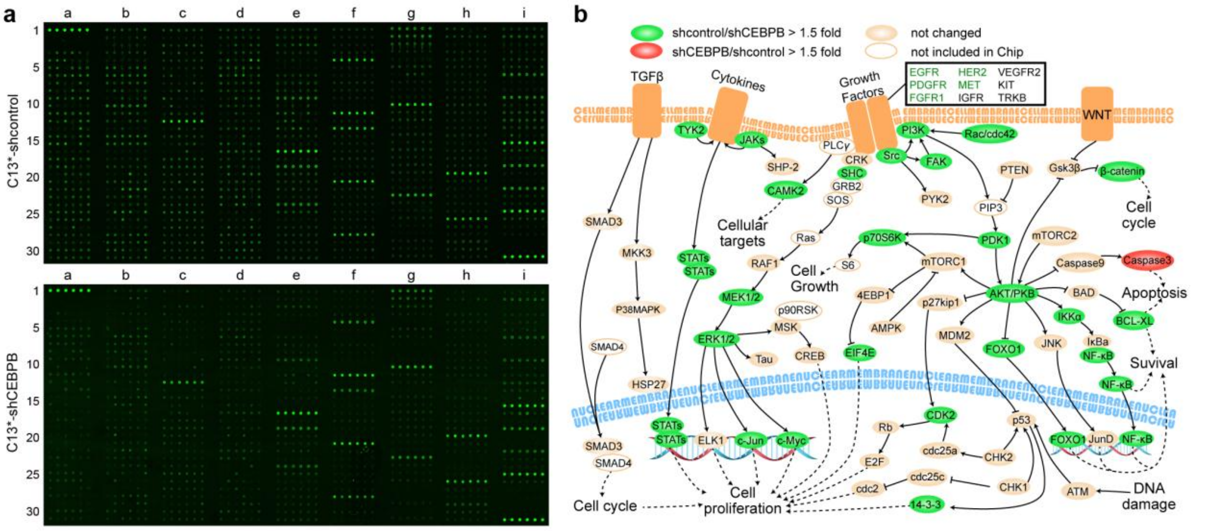

c Cancer Signaling Phospho Antibody Array (PCS248) Map:

	a	b	c	d	e	f	g	h	i
1	Positive Marker	pSTAT6 (Thr645)	pIKK α (Thr23)	pPDGFRβ (Tyr751)	pSmad3 (Ser425)	un-pJunB (79)	un-pChk2 (68)	un-pcdc25A (75)	un-pp70 S6 Kinase (424)
2	Empty	pCREB (Ser133)	pRb (Ser780)	peIF4E (Ser209)	pTrk B (Tyr515)	un-pJunB (259)	un-pp53 (315)	un-pSrc (529)	un-pCaMKII (286)
3	Empty	pAkt (Ser473)	pCDK2 (Thr160)	pMet (Tyr1349)	pCrkII (Tyr221)	un-pJunD (255)	un-pTau (404)	un-pMEK1 (221)	un-pMEK1 (291)
4	pGSK3 β (Ser9)	pAkt (Thr308)	pcdc25A (Ser75)	pFGFR1 (Tyr154)	peEF2K (Ser366)	un-pMyc (58)	un-pβCatenin (41/45)	un-pIkB-a (42)	un-p14-3-3 zeta (58)
5	pc-Jun (Ser73)	pPTEN (Ser380/Thr382/383)	pChk1 (Ser280)	pc-Kit (Tyr721)	pFak (Tyr397)	un-pMyc (358)	un-pICAM-1 (512)	un-pSTAT1 (727)	un-pAMPK1 (174)
6	pElk-1 (Ser383)	pFAK (Tyr861)	pChk1 (Ser317)	pBRCA1 (Ser1423)	pCaspase 9 (Thr125)	un-pMyc (373)	un-pRb (780)	un-pMSK1 (376)	un-pHSP90B (254)
7	pPDK1 (Ser241)	pIntegrin β3 (Tyr773)	pMKK3 (Ser189)	pCDC2 (Tyr15)	pMDM2 (Ser166)	un-pSTAT1 (701)	un-pCDK2 (160)	un-pRac1/cdc42 (71)	un-pIkB-e (22)
8	pRaf1 (Ser259)	pChk2 (Thr68)	pChk2 (Ser516)	pERK1/2 (Thr202)	pCaspase 9 (Tyr153)	un-pSTAT3 (705)	un-pCaveolin-1 (14)	un-pMEK1 (217)	un-pKeratin 18 (33)
9	pGSK3 α (Ser21)	pBCL-2 (Thr56)	pTYK2 (Tyr1054)	pERK1/2 (Tyr204)	pCaspase 9 (Ser144)	un-pSTAT3 (727)	un-pChk1 (280)	un-pFAK (397)	un-pMyc (62)
10	pMEK-2 (Thr394)	pBCL-2 (Ser70)	pJAK1 (Tyr1022)	pHSP27 (Ser78)	pCaspase 9 (Ser196)	un-pSTAT4 (693)	un-pChk1 (317)	un-pPyk2 (402)	un-pShc (349)
11	pNFkB-p65 (Thr254)	pBCL-XL (Ser62)	pJAK2 (Tyr221)	pSAPK/JNK (Thr183)	pCaspase-3 (Ser150)	un-pSTAT5A (694)	un-pSrc (418)	un-pNFkB-p65 (529)	un-pSHP-2 (580)
12	pNFkB2 (Ser865)	pBAD (Ser112)	pJAK2 (Tyr1007)	pNFkB1 (Ser932)	pERK3 (Ser189)	un-pSTAT5A (780)	un-pMKK3 (189)	un-pβCatenin (37)	un-pTrk B (515)
13	pNFkB2 (Ser869)	pBAD (Ser136)	pIkB-a (Ser32/36)	pP38 MAPK (Thr180)	pERK8 (Thr175/Tyr177)	un-pSTAT6 (641)	un-pChk2 (516)	un-pmTOR (2448)	un-pCrkII (221)
14	pNFkB1 (Ser893)	pBAD (Ser155)	pSrc (Tyr529)	pP38 MAPK (Tyr182)	pPI3K p85 (Tyr467/199)	un-pSTAT6 (645)	un-pTYK2 (1054)	un-p4E-BP1 (36)	un-peEF2K (366)
15	pNFkB1 (Ser907)	pESR1 (Ser167)	pMEK1 (Ser221)	pHSF1 (Ser303)	pPI3K p85 (Tyr607)	un-pCREB (133)	un-pJAK1 (1022)	un-pPDGFRβ (751)	un-pCaspase 9 (125)
16	pRel (Ser503)	pHER2 (Tyr877)	pIkB-a (Tyr42)	pEGFR (Tyr1110)	un-pGSK3 β (9)	un-pAkt (473)	un-pJAK2 (221)	un-peIF4E (209)	un-pMDM2 (166)
17	pc-Jun (Thr239)	pICAM-1 (Tyr512)	pSTAT1 (Ser727)	pHistone H2A.X (Ser139)	un-pc-Jun (73)	un-pAkt (308)	un-pJAK2 (1007)	un-pMet (1349)	un-pCaspase 9 (153)
18	pc-Jun (Ser243)	pVEGFR2 (Tyr951)	pHDAC8 (Ser39)	peIF2a (Ser51)	un-pElk-1 (383)	un-pPTEN (380/382/383)	un-pIkB-a (32/36)	un-pFGFR1 (154)	un-pCaspase 9 (144)
19	pJunB (Ser79)	pIGF-1R (Tyr1161)	pHSP27 (Ser15)	pIntegrin β3 (Tyr785)	un-pPDK1 (241)	un-pBCL-2 (56)	un-pFKHR (256)	un-pc-Kit (721)	un-pCaspase 9 (196)
20	pJunB (Ser259)	pCaveolin-1 (Tyr14)	pMSK1 (Ser376)	pp70 S6 Kinase (Ser424)	un-pRaf1 (259)	un-pBCL-2 (70)	un-pBRCA1 (1524)	un-pBRCA1 (1423)	un-pCaspase-3 (150)
21	pJunD (Ser255)	pSrc (Tyr418)	pRac1/cdc42 (Ser71)	pCaMKII (Thr286)	un-pGSK3 α (21)	un-pBCL-XL (62)	un-pHDAC8 (39)	un-pCDC2 (15)	un-pERK3 (189)
22	pMyc (Thr58)	pp53 (Ser6)	pMEK1 (Ser217)	pMEK1 (Thr291)	un-pMEK-2 (394)	un-pBAD (112)	un-pHSP27 (15)	un-pERK1/2 (202)	un-pERK8 (175/177)
23	pMyc (Thr358)	pp53 (Ser315)	pp21Cip1 (Thr145)	p14-3-3 zeta (Ser58)	un-pNFkB-p65 (254)	un-pBAD (136)	un-pcdc25C (216)	un-pERK1/2 (204)	un-pPI3K p85 (467/199)
24	pMyc (Ser373)	pTau (Ser404)	pp27Kip1 (Ser10)	pAMPK1 (Thr174)	un-pNFkB2 (865)	un-pBAD (155)	un-pChk1 (345)	un-pHSP27 (78)	un-pPI3K p85 (607)
25	pSTAT1 (Tyr701)	pFKHR (Ser256)	pp27Kip1 (Thr187)	pHSP90B (Ser254)	un-pNFkB2 (869)	un-pESR1 (167)	un-pATM (1981)	un-pSAPK/JNK (183)	β-Actin
26	pSTAT3 (Tyr705)	pβCatenin (Thr41/Ser45)	pPyk2 (Tyr402)	pIkB-b (Ser23)	un-pNFkB1 (337)	un-pHER2 (877)	un-pFAK (925)	un-pP38 MAPK (182)	GAPDH
27	pSTAT3 (Ser727)	pBRCA1 (Ser1524)	pNFkB-p65 (Ser529)	pIkB-e (Ser22)	un-pNFkB1 (893)	un-pFAK (861)	un-pp21Cip1 (145)	un-pHSF1 (303)	Negative control
28	pSTAT4 (Tyr693)	pcdc25C (Ser216)	pβCatenin (Ser33)	pKeratin 18 (Ser33)	un-pNFkB1 (907)	un-pVEGFR2 (951)	un-pp27Kip1 (10)	un-pEGFR (1110)	Negative control
29	pSTAT5A (Tyr694)	pChk1 (Ser345)	pβCatenin (Ser37)	pMyc (Ser62)	un-pRel (503)	un-pIGF-1R (1161)	un-pp27Kip1 (187)	un-pHistone H2A.X (139)	Empty
30	pSTAT5A (Ser780)	pFAK (Tyr925)	pmTOR (Ser2448)	pShc (Tyr349)	un-pc-Jun (239)	un-pIntegrin β3 (773)	un-pAkt2 (474)	un-peIF2a (51)	Empty
31	pSTAT6 (Tyr641)	pAkt2 (Ser474)	p4E-BP1 (Thr36)	pSHP-2 (Tyr580)	un-pc-Jun (243)	un-pp53 (6)	un-pIKK a (23)	un-pIntegrin β3 (785)	Positive Marker

Supplementary Figure 21. Analysis of phospho-antibody array. a, C13*-shcontrol and C13*-NshCEBPB cells were treated with cisplatin (50 μM) for 24h, and the phosphorylation states of proteins were then detected using antibody-based arrays. **b**, Graphic of the pathways involved in the phospho-antibody array. Proteins with decreased Phospho Ratio are in green; proteins with increased Phospho Ratio are in red. Lines correspond to physical interactions. Solid lines imply direct relationships and dotted lines imply indirect relationships. Functional relationships include post-translational modifications, transcription regulation, proteolysis or co-expression. Arrows indicate promotion; flat arrowheads indicate inhibition. **c**, Antibody array map. The locations of antibodies on the array are indicated.

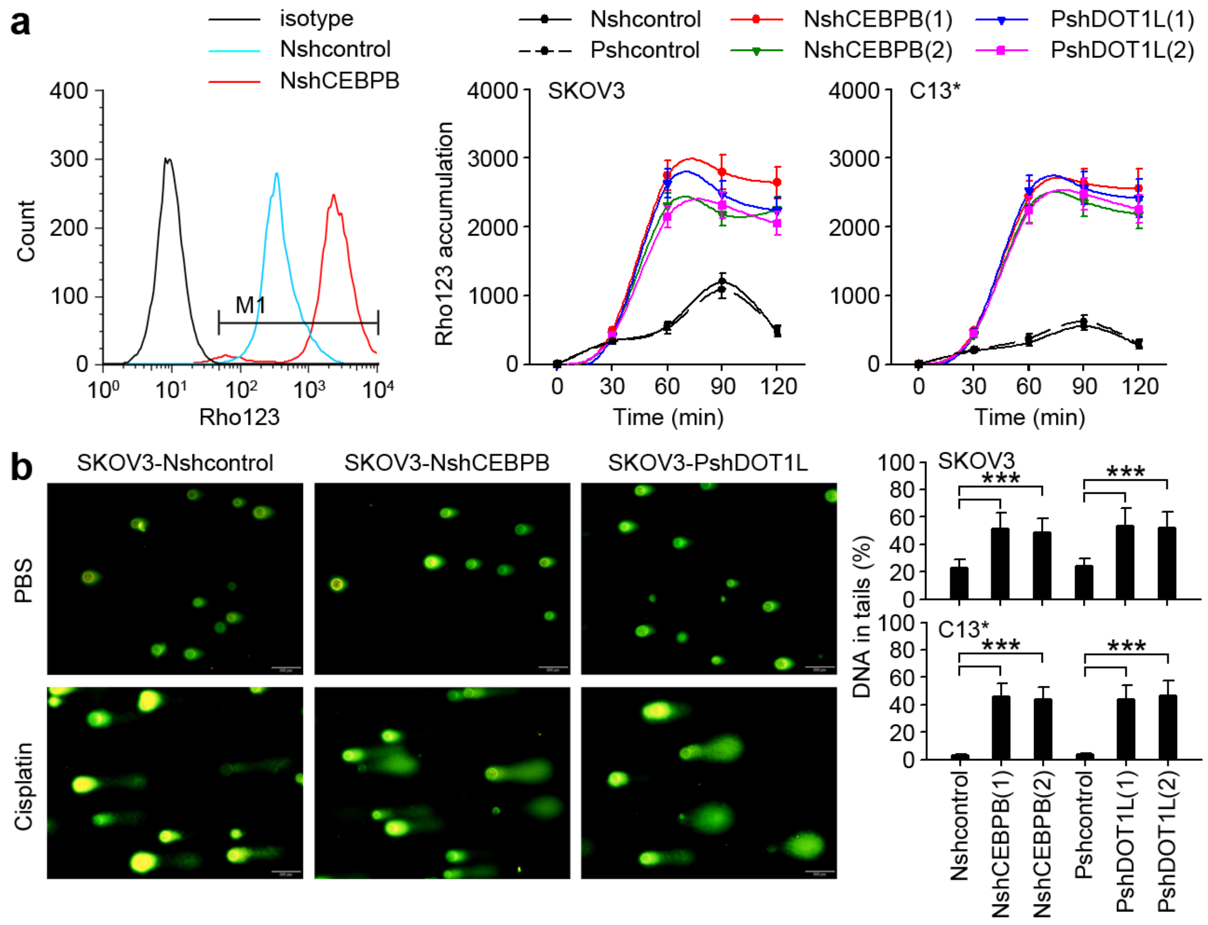

Supplementary Figure 22. Knocking down the expression of C/EBPβ or DOT1L enhance intercellular accumulation of drugs and cisplatin-induced DNA damage. a, Drug accumulation assay. The indicated cells were treated with 1 μM rhodamine123 (Rho123) for a series of times, the cellular accumulation of Rho123 was determined by flow cytometric analysis. **b**, The indicated cells were treated with cisplatin (50 μM) for 24 h and analyzed by alkaline comet assay. Representative images are shown (left panel). The average percentage of the DNA in tails was measured (right panel). ***, $P < 0.001$.

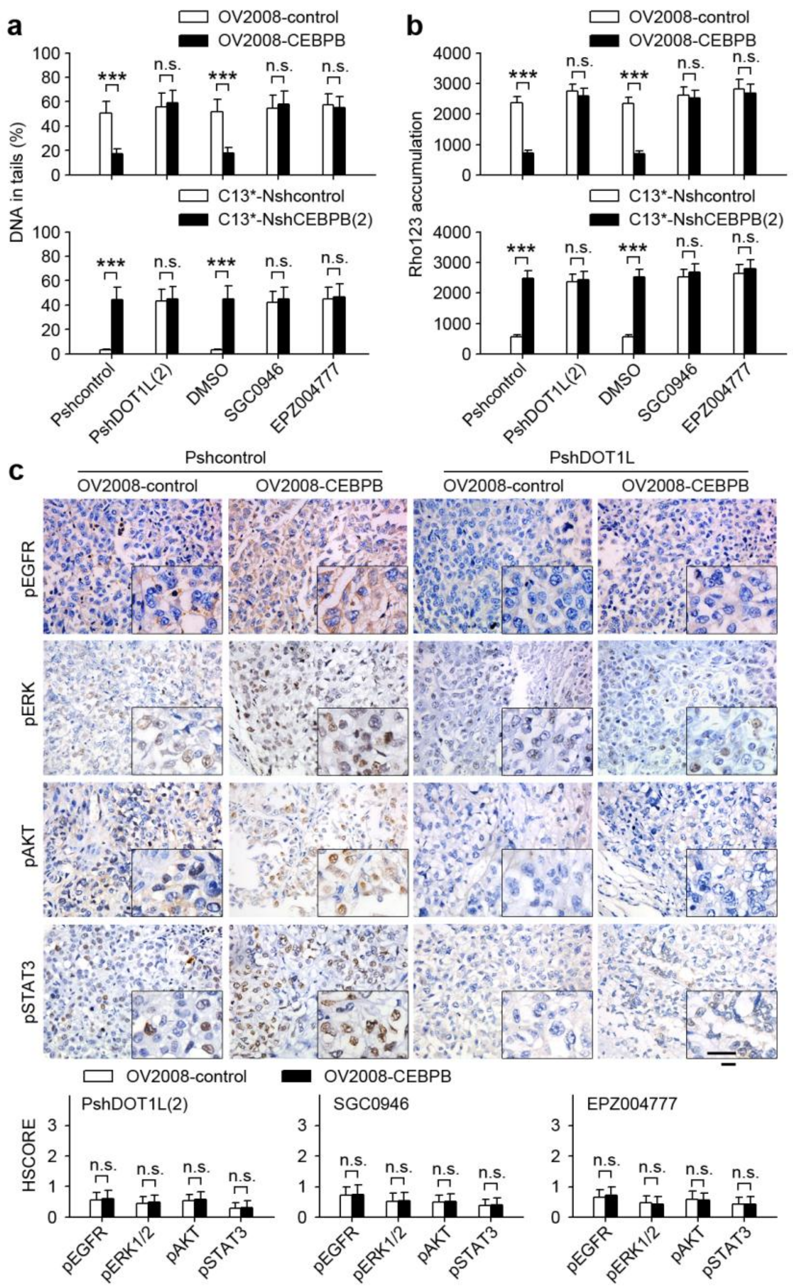

Supplementary Figure 23. The promotional effect of C/EBPβ on cisplatin-resistance capacity is mediated by DOT1L. a, b, For DOT1L inhibitors treatment, cells were pre-cultured in the presence of SGC0946 (5 μM) or EPZ004777 (5 μM) for up to 12 days. The indicated cells were treated with cisplatin (50 μM) for 24 h, and then analyzed by alkaline comet assay. The average percentage of the DNA in tails was measured (**a**). The indicated cells were treated with 1 μM Rho123 for 1 h, and then the cellular accumulation of Rho123 was determined by flow cytometric analysis (**b**). **c**, One week after orthotopical inoculation with the indicated cells, the mice were treated with 5 mg/kg cisplatin intraperitoneally every 4 days; for DOT1L inhibitors treatment, 4 mg/kg SGC0946 or EPZ004777 were injected intraperitoneally every 4 days. The mice were euthanized at 6 weeks after tumor inoculation. IHC analysis of the phosphorylated EGFR, ERK1/2, AKT and STAT3 on the xenograft tumor sections, and the HSCOREs were calculated (n = 10 per group). ***, $P < 0.001$. n.s. indicates "not significant".

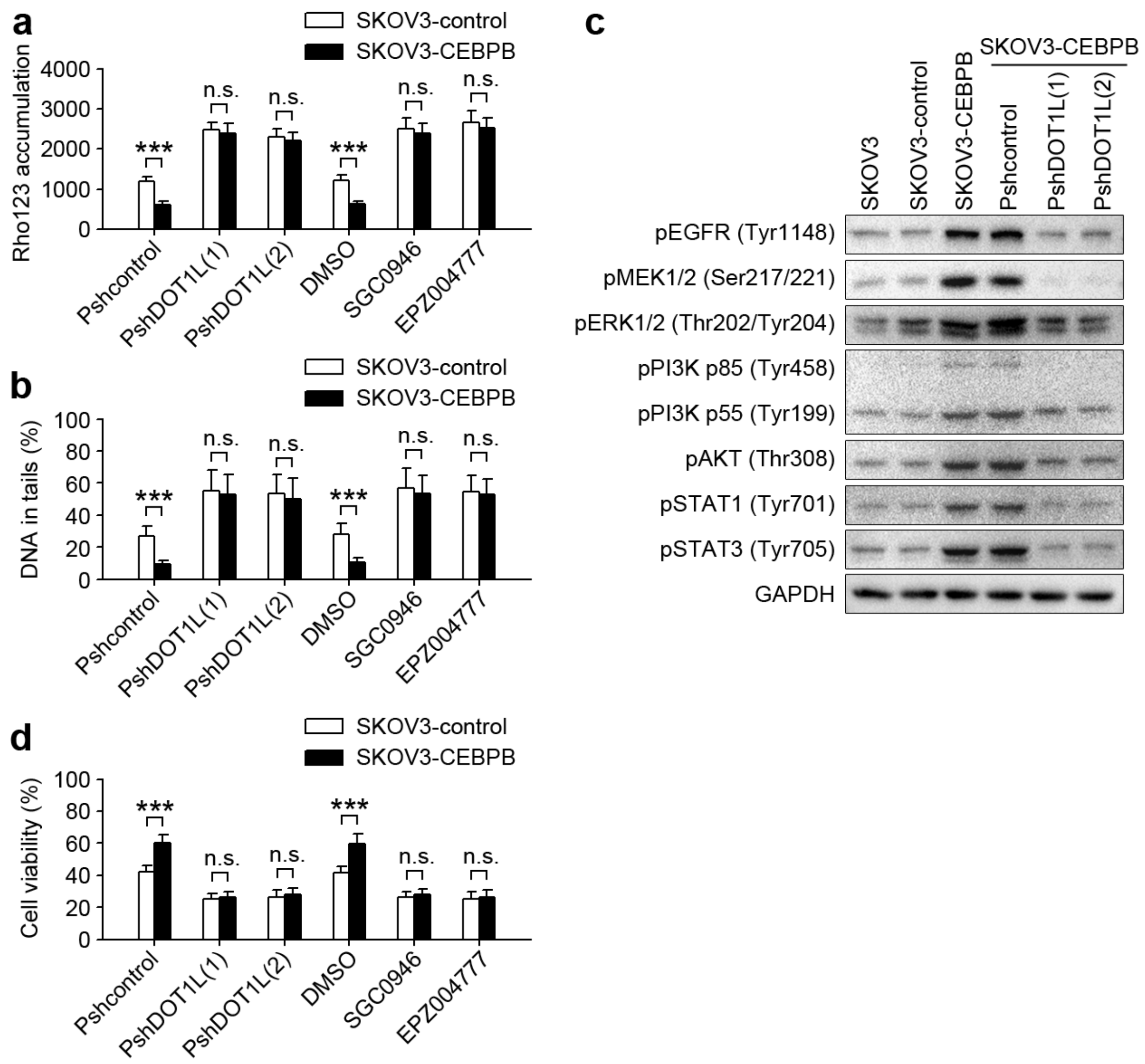

Supplementary Figure 24. The promotional effect of C/EBPβ on cisplatin resistance of SKOV3 cells is mediated by DOT1L. For DOT1L inhibitors treatment, cells were pre-cultured in the presence of SGC0946 (5 μM) or EPZ004777 (5 μM) for up to 12 days., and then used in the following experiments. **a**, Drug accumulation assay. The indicated cells were treated with 1 μM Rho123 for 1 h, the cellular accumulation of Rho123 was determined by flow cytometric analysis. **b**, The indicated cells were treated with cisplatin (50 μM) for 24 h and analyzed by alkaline comet assay. The average percentage of the DNA in tails was measured. **c**, The indicated cells were treated with cisplatin (50 μM) for 24 h, the phosphorylation levels of proteins were detected by western blotting. **d**, Cell viability of the indicated cell lines was determined by the CCK8 assay (at 36 h following 50 μM cisplatin treatment). Uncropped images of blots are shown in Supplementary Fig. 26. ***, $P < 0.001$. n.s. indicates “not significant”.

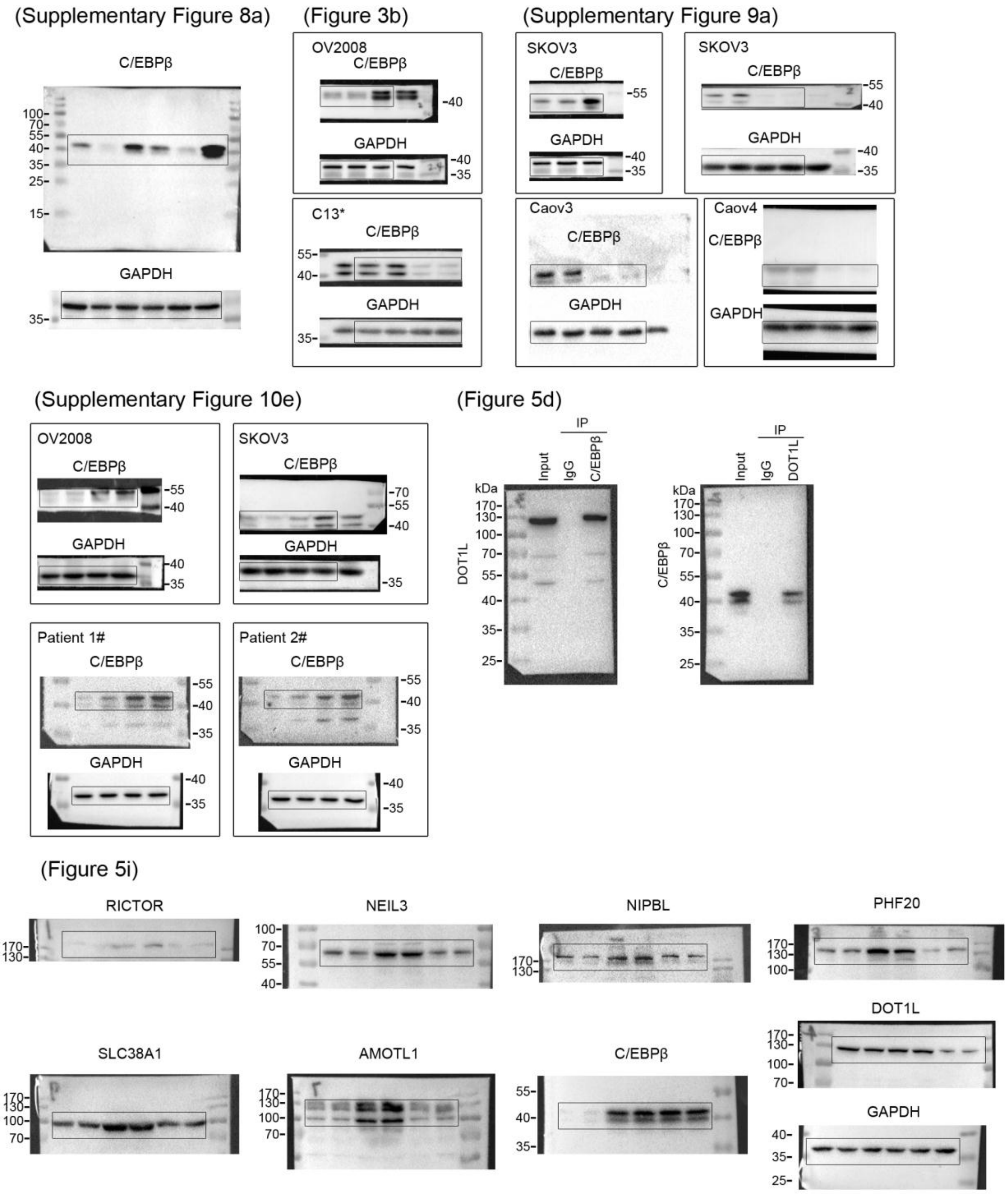

Supplementary Figure 25. Uncropped gel images (1). Uncropped gel images corresponding to Supplementary Figure 8a, Figure 3b, Supplementary Figure 9a, Supplementary Figure 10e, Figure 5d and Figure 5i.

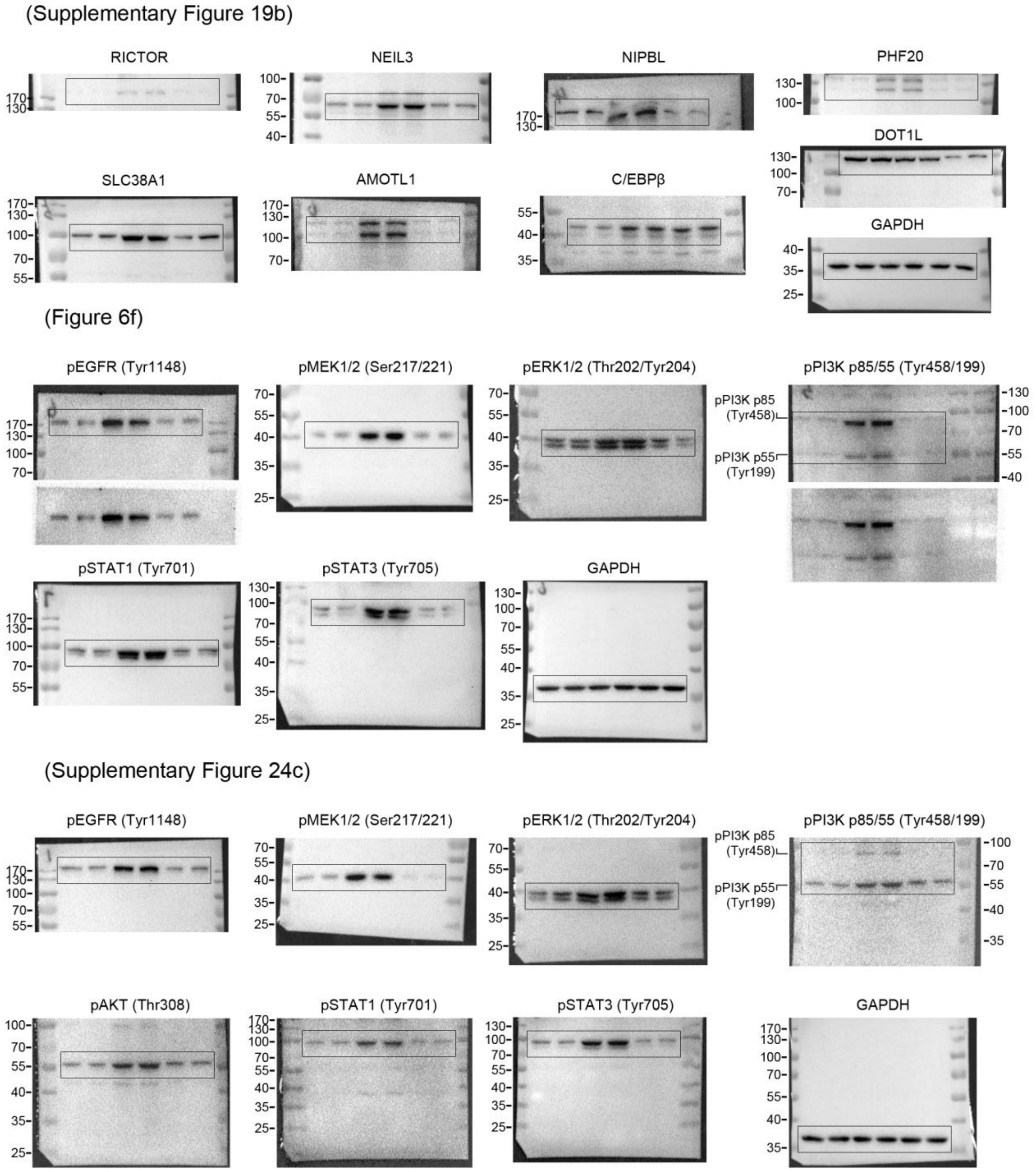

Supplementary Figure 26. Uncropped gel images (2). Uncropped gel images corresponding to Supplementary Figure 19b, Figure 6f and Supplementary figure 24c.

Supplementary References

1. Golding, S. E. *et al.* Pro-survival AKT and ERK signaling from EGFR and mutant EGFRvIII enhances DNA double-strand break repair in human glioma cells. *Cancer Biol Ther* **8**, 730-738 (2009).
2. Wang, Y. *et al.* Inhibition of both EGFR and IGF1R sensitized prostate cancer cells to radiation by synergistic suppression of DNA homologous recombination repair. *PLoS One* **8**, e68784, doi:10.1371/journal.pone.0068784 (2013).
3. Rohwer, N., Zasada, C., Kempa, S. & Cramer, T. The growing complexity of HIF-1 alpha's role in tumorigenesis: DNA repair and beyond. *Oncogene* **32**, 3569-3576 (2013).
4. Wirthner, R., Wrann, S., Balamurugan, K., Wenger, R. H. & Stiehl, D. P. Impaired DNA double-strand break repair contributes to chemoresistance in HIF-1 alpha-deficient mouse embryonic fibroblasts. *Carcinogenesis* **29**, 2306-2316 (2008).
5. Wan, G. *et al.* DNA-damage-induced nuclear export of precursor microRNAs is regulated by the ATM-AKT pathway. *Cell reports* **3**, 2100-2112 (2013).
6. Schmidt, F., Kunze, M., Loock, A. C. & Dobbelstein, M. Screening analysis of ubiquitin ligases reveals G2E3 as a potential target for chemosensitizing cancer cells. *Oncotarget* **6**, 617-632 (2015).
7. Kong, X. *et al.* Distinct functions of human cohesin-SA1 and cohesin-SA2 in double-strand break repair. *Mol Cell Biol* **34**, 685-698 (2014).
8. Enervald, E. *et al.* A regulatory role for the cohesin loader NIPBL in nonhomologous end joining during immunoglobulin class switch recombination. *J Exp Med* **210**, 2503-2513 (2013).
9. Szumiel, I. Epidermal growth factor receptor and DNA double strand break repair: the cell's self-defence. *Cell Signal* **18**, 1537-1548 (2006).
10. Lemaitre, C. *et al.* The nucleoporin 153, a novel factor in double-strand break repair and DNA damage response. *Oncogene* **31**, 4803-4809 (2012).
11. Rolseth, V. *et al.* Loss of Neil3, the major DNA glycosylase activity for removal of hydantoins in single stranded DNA, reduces cellular proliferation and sensitizes cells to genotoxic stress. *Biochim Biophys Acta* **1833**, 1157-1164 (2013).
12. Fujioka, T. & Ui, M. Involvement of insulin receptor substrates in epidermal growth factor induced activation of phosphatidylinositol 3-kinase in rat hepatocyte primary culture. *Eur J Biochem* **268**, 25-34 (2001).
13. Schulze, W. X., Deng, L. & Mann, M. Phosphotyrosine interactome of the ErbB-receptor kinase family. *Mol Syst Biol* **1**, 2005 0008 (2005).
14. Park, J. H. *et al.* Loss of Mel-18 induces tumor angiogenesis through enhancing the activity and expression of HIF-1alpha mediated by the PTEN/PI3K/Akt pathway. *Oncogene* **30**, 4578-4589 (2011).
15. Blancher, C., Moore, J. W., Robertson, N. & Harris, A. L. Effects of ras and von Hippel-Lindau (VHL) gene mutations on hypoxia-inducible factor (HIF)-1alpha, HIF-2alpha, and vascular endothelial growth factor expression and their regulation by the phosphatidylinositol 3'-kinase/Akt signaling pathway. *Cancer Res* **61**, 7349-7355 (2001).
16. Im-aram, A. *et al.* The mTORC2 component rictor contributes to cisplatin resistance in human ovarian cancer cells. *PLoS One* **8**, e75455, doi:10.1371/journal.pone.0075455 (2013).

17. Wang, K. *et al.* Activation of SNAT1/SLC38A1 in human breast cancer: correlation with p-Akt overexpression. *BMC cancer* **13**, 343 (2013).
18. Lin, C. M. *et al.* Inhibition of Transient Receptor Potential Melastain 7 Enhances Apoptosis Induced by TRAIL in PC-3 cells. *Asian Pacific journal of cancer prevention : APJCP* **16**, 4469-4475 (2015).
19. Fang, L. *et al.* TRPM7 channel regulates PDGF-BB-induced proliferation of hepatic stellate cells via PI3K and ERK pathways. *Toxicology and applied pharmacology* **272**, 713-725 (2013).
20. West, N. R., Murray, J. I. & Watson, P. H. Oncostatin-M promotes phenotypic changes associated with mesenchymal and stem cell-like differentiation in breast cancer. *Oncogene* **33**, 1485-1494 (2014).
21. Ratushny, V., Astsaturov, I., Burtness, B. A., Golemis, E. A. & Silverman, J. S. Targeting EGFR resistance networks in head and neck cancer. *Cell Signal* **21**, 1255-1268 (2009).
22. Kohsaka, S. *et al.* Epiregulin enhances tumorigenicity by activating the ERK/MAPK pathway in glioblastoma. *Neuro-Oncology* **16**, 960-970 (2014).
23. Li, L. *et al.* The requirement of extracellular signal-related protein kinase pathway in the activation of hypoxia inducible factor 1 alpha in the developing rat brain after hypoxia-ischemia. *Acta Neuropathol* **115**, 297-303 (2008).
24. Gordan, J. D. *et al.* HIF-alpha effects on c-Myc distinguish two subtypes of sporadic VHL-deficient clear cell renal carcinoma. *Cancer Cell* **14**, 435-446 (2008).
25. Das, G., Shiras, A., Shanmuganandam, K. & Shastry, P. Rictor regulates MMP-9 activity and invasion through Raf-1-MEK-ERK signaling pathway in glioma cells. *Molecular carcinogenesis* **50**, 412-423 (2011).
26. Cheng, C. I., Chen, P. H., Lin, Y. C. & Kao, Y. H. High glucose activates Raw264.7 macrophages through RhoA kinase-mediated signaling pathway. *Cell Signal* **27**, 283-292 (2015).
27. Li, F. *et al.* RhoA modulates functional and physical interaction between ROCK1 and Erk1/2 in selenite-induced apoptosis of leukaemia cells. *Cell Death Dis* **4**, e708, doi:10.1038/cddis.2013.243 (2013).
28. Kuper, C., Beck, F. X. & Neuhofer, W. NFAT5-mediated expression of S100A4 contributes to proliferation and migration of renal carcinoma cells. *Frontiers in physiology* **5**, 293 (2014).
29. Tsai, T. T. *et al.* MEK/ERK signaling controls osmoregulation of nucleus pulposus cells of the intervertebral disc by transactivation of TonEBP/OREBP. *J Bone Miner Res* **22**, 965-974 (2007).
30. Beigel, F. *et al.* Oncostatin M mediates STAT3-dependent intestinal epithelial restitution via increased cell proliferation, decreased apoptosis and upregulation of SERPIN family members. *PLoS One* **9**, e93498, doi:10.1371/journal.pone.0093498 (2014).
31. Grandis, J. R. *et al.* Constitutive activation of Stat3 signaling abrogates apoptosis in squamous cell carcinogenesis in vivo. *Proc Natl Acad Sci U S A* **97**, 4227-4232 (2000).
32. Yue, P. *et al.* Hyperactive EGF receptor, Jaks and Stat3 signaling promote enhanced colony-forming ability, motility and migration of cisplatin-resistant ovarian cancer cells. *Oncogene* **31**, 2309-2322 (2012).
33. Gao, W. *et al.* Hypoxia and STAT3 signalling interactions regulate pro-inflammatory

pathways in rheumatoid arthritis. *Ann Rheum Dis* **74**, 1275-1283 (2015).
34. Xu, W. *et al.* Enhanced expression of cohesin loading factor NIPBL confers poor prognosis and chemotherapy resistance in non-small cell lung cancer. *J Transl Med* **13**, 153 (2015).
35. Fossey, S. L., Bear, M. D., Kisseberth, W. C., Pennell, M. & London, C. A. Oncostatin M promotes STAT3 activation, VEGF production, and invasion in osteosarcoma cell lines. *BMC cancer* **11**, 125 (2011).
36. Liu, M., Inoue, K., Leng, T., Guo, S. & Xiong, Z. G. TRPM7 channels regulate glioma stem cell through STAT3 and Notch signaling pathways. *Cell Signal* **26**, 2773-2781 (2014).
37. Rohwer, N. *et al.* Hypoxia-inducible factor 1alpha determines gastric cancer chemosensitivity via modulation of p53 and NF-kappaB. *PLoS One* **5**, e12038, doi:10.1371/journal.pone.0012038 (2010).
38. Zhang, T. *et al.* PHF20 regulates NF-kappaB signalling by disrupting recruitment of PP2A to p65. *Nat Commun* **4**, 2062 (2013).
39. Ai, L., Skehan, R. R., Saydi, J., Lin, T. & Brown, K. D. Ataxia-Telangiectasia, Mutated (ATM)/Nuclear Factor kappa light chain enhancer of activated B cells (NFkappaB) signaling controls basal and DNA damage-induced transglutaminase 2 expression. *J Biol Chem* **287**, 18330-18341 (2012).
40. Yang, X. *et al.* Mechanism of fibrotic cardiomyopathy in mice expressing truncated Rho-associated coiled-coil protein kinase 1. *FASEB J* **26**, 2105-2116 (2012).
41. Zhu, L. *et al.* Salidroside ameliorates arthritis-induced brain cognition deficits by regulating Rho/ROCK/NF-kappaB pathway. *Neuropharmacology* **103**, 134-142 (2016).
42. Porcelli, L. *et al.* The EGFR pathway regulates BCRP expression in NSCLC cells: role of erlotinib. *Curr Drug Targets* **15**, 1322-1330 (2014).
43. Comerford, K. M. *et al.* Hypoxia-inducible factor-1-dependent regulation of the multidrug resistance (MDR1) gene. *Cancer Res* **62**, 3387-3394 (2002).
44. Liu, L. *et al.* Hypoxia-inducible factor-1 alpha contributes to hypoxia-induced chemoresistance in gastric cancer. *Cancer science* **99**, 121-128 (2008).
45. Galluzzi, L. *et al.* Molecular mechanisms of cisplatin resistance. *Oncogene* **31**, 1869-1883 (2012).
46. Borst, P., Evers, R., Kool, M. & Wijnholds, J. A family of drug transporters: the multidrug resistance-associated proteins. *J Natl Cancer Inst* **92**, 1295-1302 (2000).
47. Young, L. C. *et al.* Expression of multidrug resistance protein-related genes in lung cancer: correlation with drug response. *Clin Cancer Res* **5**, 673-680 (1999).
48. Lo, H. W. & Ali-Osman, F. Genetic polymorphism and function of glutathione S-transferases in tumor drug resistance. *Current opinion in pharmacology* **7**, 367-374 (2007).
49. Fu, J. *et al.* TGM2 inhibition attenuates ID1 expression in CD44-high glioma-initiating cells. *Neuro Oncol* **15**, 1353-1365 (2013).
50. Wang, M. D. *et al.* Acetyl-coenzyme A carboxylase alpha promotion of glucose-mediated fatty acid synthesis enhances survival of hepatocellular carcinoma in mice and patients. *Hepatology* **63**, 1272-1286 (2016).
51. Farooqui, M. *et al.* Epiregulin contributes to breast tumorigenesis through regulating

matrix metalloproteinase 1 and promoting cell survival. *Molecular cancer* **14**, 138 (2015).
52. Isikbay, M. *et al.* Glucocorticoid receptor activity contributes to resistance to androgen-targeted therapy in prostate cancer. *Hormones & cancer* **5**, 72-89 (2014).
53. Oka, T., Schmitt, A. P. & Sudol, M. Opposing roles of angiomotin-like-1 and zona occludens-2 on pro-apoptotic function of YAP. *Oncogene* **31**, 128-134 (2012).
54. Schackmann, R. C. *et al.* Cytosolic p120-catenin regulates growth of metastatic lobular carcinoma through Rock1-mediated anoikis resistance. *J Clin Invest* **121**, 3176-3188 (2011).
55. Hao, J. *et al.* Effects of lentivirus-mediated HIF-1alpha knockdown on hypoxia-related cisplatin resistance and their dependence on p53 status in fibrosarcoma cells. *Cancer gene therapy* **15**, 449-455 (2008).
56. Rohwer, N. & Cramer, T. Hypoxia-mediated drug resistance: novel insights on the functional interaction of HIFs and cell death pathways. *Drug Resist Updat* **14**, 191-201 (2011).
57. Li, Y. *et al.* PKB-mediated PHF20 phosphorylation on Ser291 is required for p53 function in DNA damage. *Cell Signal* **25**, 74-84 (2013).
58. Koeppel, M. *et al.* The novel p53 target gene IRF2BP2 participates in cell survival during the p53 stress response. *Nucleic Acids Res* **37**, 322-335 (2009).
59. DeClerck, K. & Elble, R. C. The role of hypoxia and acidosis in promoting metastasis and resistance to chemotherapy. *Frontiers in bioscience* **15**, 213-225 (2010).
60. Wysocki, P. J. mTOR in renal cell cancer: modulator of tumor biology and therapeutic target. *Expert Rev Mol Diagn* **9**, 231-241 (2009).
61. Auf, G. *et al.* High epiregulin expression in human U87 glioma cells relies on IRE1alpha and promotes autocrine growth through EGF receptor. *BMC cancer* **13**, 597 (2013).
62. Wang, J. *et al.* TRPM7 is required for ovarian cancer cell growth, migration and invasion. *Biochem Biophys Res Commun* **454**, 547-553 (2014).
63. Guo, K. & Jin, F. NFAT5 promotes proliferation and migration of lung adenocarcinoma cells in part through regulating AQP5 expression. *Biochem Biophys Res Commun* **465**, 644-649 (2015).
64. Liu, Q. *et al.* Berberine induces senescence of human glioblastoma cells by downregulating the EGFR-MEK-ERK signaling pathway. *Mol Cancer Ther* **14**, 355-363 (2015).
65. Granados, M. L., Hudson, L. G. & Samudio-Ruiz, S. L. Contributions of the Epidermal Growth Factor Receptor to Acquisition of Platinum Resistance in Ovarian Cancer Cells. *PLoS One* **10**, e0136893, doi:10.1371/journal.pone.0136893 (2015).
66. Ohta, T. *et al.* Inhibition of the Rho/ROCK pathway enhances the efficacy of cisplatin through the blockage of hypoxia-inducible factor-1alpha in human ovarian cancer cells. *Cancer Biol Ther* **13**, 25-33 (2012).
