# Supplementary material for: C/EBPβ enhances platinum resistance of ovarian cancer cells by reprogramming H3K79 methylation
Source: Nat Commun. 2018 Apr 30;9:1739. doi: 10.1038/s41467-018-03590-5 (PMC5928165; doi:10.1038/s41467-018-03590-5)
Supplement: Supplementary file 3 — Description of Additional Supplementary Files(PDF 177 kb) [file 41467_2018_3590_MOESM3_ESM.pdf]

Description of Additional Supplementary Files

File Name: Supplementary Data 1
Description: H3K79me2/me3 ChIP-seq anotated decreased peaks in C/EBPβ knockdown cells.

File Name: Supplementary Data 2
Description: C/EBPβ ChIP-seq anotated peaks.

File Name: Supplementary Data 3
Description: H3K9me3 ChIP-seq anotated increased peaks in C/EBPβ knockdown cells.

File Name: Supplementary Data 4
Description: Lists of genes up and down regulated in C/EBPβ knockdown cells.

File Name: Supplementary Data 5
Description: Ingenuity pathway analysis (IPA) of the differentially expressed genes in C/EBPβ knockdown cells.

File Name: Supplementary Data 6
Description: KEGG pathway analysis of the differentially expressed genes in C/EBPβ knockdown cells.

File Name: Supplementary Data 7
Description: DOT1L ChIP-seq anotated peaks.

File Name: Supplementary Data 8
Description: Medium-scale screening analysis of cisplatin-resistance genes of ovarian cancer.

File Name: Supplementary Data 9
Description: Knockdown of C/EBPβ in C13* cells decreases phosphorylation and activation of survival signals.

File Name: Supplementary Data 10
Description: Primers for ChIP-qPCR.

File Name: Supplementary Data 11
Description: Primers for RT-qPCR.
