# Supplementary material for: C/EBPβ enhances platinum resistance of ovarian cancer cells by reprogramming H3K79 methylation
Source: Nat Commun. 2018 Apr 30;9:1739. doi: 10.1038/s41467-018-03590-5 (PMC5928165; doi:10.1038/s41467-018-03590-5)
Supplement: Supplementary file 13 — Supplementary Data 10(DOC 141 kb) [file 41467_2018_3590_MOESM13_ESM.doc]

**Supplementary Data 10. Primers for ChIP**-qPCR

| Gene | Sense primers | Antisense primers |
| --- | --- | --- |
| ABCC1_p1 | GGGGTGTCTCTCTGCTTCTG | CCGTTGGTCACGGAGTATTT |
| ABCC1_p2 | GGATTCCCCTTTTCTTCAGC | AGGCAGACAGTAGCCCAGAA |
| ABCC1_p3 | GAATTCCTCTTGGGGCTAGG | AAAACCAAAGCCACACTTGG |
| ABCC1_p4 | GAGCCACCATACCCAGTCAT | CTGGGCCAGCTCACTTTTAG |
| ABCC1_p5 | CGCAATAGTTTGGCTCACAA | TTACAGGCATGAGCCATCAC |
| ABCC1_p6 | CTGCCTGGTGACCTCTCTTC | AGAGCTCCTGGGATGACAGA |
| ABCC1_p7 | CTGGAAGAGCTGCCATGAAT | AGGACTAGCGTCAGCTCCAC |
| ABCC3_p1 | CTGCCTCAGGTTAGGACTCG | TAAGAAGGGTGGGACACTGC |
| ABCC3_p2 | TTGCCTCATGAGCACACTTC | GGGCACAACTACCCTCTTGA |
| ABCC3_p3 | CTGAGGGCCAGAGAGATACG | GCCCAGTGTTTCAAGACCAT |
| ABCC3_p4 | TGTGCCTGTAGTCCCAGCTA | CAGGCTGGAATGCAGTGATA |
| ACACA_p1 | AACTCCAGCACTTTGGGAGA | CTGCCTGGTTCAAGCAACTC |
| ACACA_p2 | TCTCCCCCTTTCACAAGAGA | TTCTCCCGGTAACTGATTCG |
| ACACA_p3 | AGAGGGTGCGTTTCAATCAG | CTCAATTTGGGCCTCTGAAG |
| ACACA_p4 | AGAAGCAGCAAGTGCAGTGA | CTCTCATTTGCTGTGCTCCA |
| ACACA_p5 | ACGAGGTCAGGAGATCGAGA | TTCACGCCATTCTTCTTCCT |
| AMOTL1_p1 | GCTTGGCCTTTGCTTACTTG | TAGGCTTGTGTCGCAATCAG |
| AMOTL1_p2 | TCAGCTTTGGAAGGCTCAAT | AAGGGCTCTGCAATTCTCAA |
| AMOTL1_p3 | GCCAGAATGCGAGAGGATAG | GTGGAGAAGCTGGGATTCAA |
| AMOTL1_p4 | AGCCCATGACTGCCAATTAC | GAGGACTGGTTGGGATTCAA |
| CASC5_p1 | CTGAAACTGATGGTGCCAGA | TGGTGGCTTACTTTTGCTTG |
| CASC5_p2 | AATATCAAGGGGCTGTGACG | TTTCGACCGAATTTCCTCTG |
| CASC5_p3 | GTGTGGACCCCAAACAAGTC | AGCCCAGAACGAAAGAGTCA |
| CASC5_p4 | ATTTCAGGCTTTGCAGGCTA | TCCGTAGGTCCTCAAACAGC |
| CASC5_p5 | CTCAAGTGATCCACCCACCT | ACACTGGTACGGCCAAACTC |
| CDC42BPA_p1 | TCCCCCTTCTATGCATTCTG | CTCGTGCAACGTAATCCTGA |
| CDC42BPA_p2 | CTGCTCAATTGTTGCCTTGA | TTTGCCCATCACAGAATTGA |
| CDC42BPA_p3 | TTGAAGGCTTGGCTCAAGTT | TGGCCAAAATCCTTCAAAAG |
| CDC42BPA_p4 | AGTGGGCAGGTCAAAGTCTG | GCCATCGCTTTGAGTTAAGG |
| DCBLD2_p1 | ACCCAGAACGCATTTCTCTG | CAAGATGCCCTGTACAGCAA |
| DCBLD2_p2 | ATAGTCACCCCCTTCCCACT | TGAGCCCGAGTAGACTGGAT |
| DCBLD2_p3 | TCACAGAATTCCTCCCAGGT | TGGCTAGATCTGCTGTGCTG |
| DCBLD2_p4 | CTTTTTGGCAAGGCCATTTA | GGTTCTTTTTCCTCCCCAAA |
| DCBLD2_p5 | TGCAGGGTGATTTCTTTTCC | CCTTATCCTTCCCACTGCAA |
| DCBLD2_p6 | CAAATGAGGGATTGCCTTGT | TCCCAAAGTGCTCGGATTAC |
| EGFR_p1 | TTTTTGGAGGAGGAGGGTTT | ATTCCAAGGACATGCTCAGG |
| EGFR_p2 | GGGACTGACAATTGCAAAGC | GGGGATAGCAGCATCAATGT |
| EGFR_p3 | GGGTGTGGAGGTGTGAAACT | TTCACATGATGCCTGCTTTC |
| EGFR_p4 | CAAGCATGTTCAGCCCACTA | TGCATCACCATACCTGGCTA |
| EGFR_p5 | GCATGACTTCAACGCACAGT | AATGTTTGTGCCTGGGTCTC |
| EGFR_p6 | ACTTGACAGGGGAAACATGC | CAAGGTCTGGGAACCACTGT |
| EREG-p1 | GCCAGTGGCAGAAAGAGAAG | GGTGGGGTAGAGGGTGAAAT |
| EREG-p2 | ATGTTTGGCCATAGGCATCT | GGCGCAACAGTTTGGACTAT |
| EREG-p3 | CTGTTGGGCAACCACTTCTT | GCCTCTTCCCTGTGAGTCAG |
| EREG-p4 | GTCGAGGGTATCTGGAGCAG | ATACCCTCAGCGATCACAGG |
| EREG-p5 | AGGCCTTTCCTGTCTGGATT | GCTGAATCAGACACCCCAAT |
| FAT1_p1 | GGAAAATGTCTTTGGGCAAG | TTGCCCTCCAAGGTTAAATG |
| FAT1_p2 | GTCTCTTCCCCATGAATTGC | GCTTCCCAATGCTCCATTTA |
| FAT1_p3 | TGTGTGGCTCTAAGCGAGTG | AACAGCATCGCCTCTCAAGT |
| FAT1_p4 | GTCACCCACTTTCGAGGTGT | ATCCAGCAAATGCAAACTCC |
| FAT1_p5 | AGAGAGCCAGCTTTCTGCTG | ACAATTGCGTCCTTGTTTCC |
| G2E3_p1 | GCCAGGCTGATCTCAAACTC | CCTCATTTTGGCACAATCCT |
| G2E3_p2 | CAACCTCTTCTGCCAACCTC | AACAGGATGGGTCAATGGAA |
| G2E3_p3 | TGAAATGCCTGAGTGGGATT | CTCGGATTTTTGCGGTACAT |
| G2E3_p4 | GGAGGATGGCATTGAACAGT | GCCCCAGTTCCATGTCTTTA |
| G2E3_p5 | TCTTGGACTCCAGCTGTCCT | GCTTGAGGCTAGGTGTTTGC |
| GFOD1_p1 | TATGGACTACGAGGGGCATC | TGCAGAATGCATCCAGAGTC |
| GFOD1_p2 | ATTTGCTCAAGGTCCCACAG | CTTTCAGGGAGCAGAGGATG |
| GFOD1_p3 | GAACATGGCAGCAGAATGAG | CTAGCTCTCCGTTGGGAATG |
| GFOD1_p4 | CTCAGAGCTCCCTCCTTCCT | ATCTACACACGTGGGGAAGC |
| GFOD1_p5 | ATGAGCCCTGCGTATACTGG | TCTGAACCCAGGACAGGAAC |
| HIF1A_p1 | TTGTCCTTTCGGGACTTGAC | CAGGTAGGATCGAGCTTTGG |
| HIF1A_p2 | GTTAAAGGGTCCCACAGCAA | AAAAGCCTGGTGTGAATTGG |
| HIF1A_p3 | TGACTGCTTCTGCTTCTTCG | TTCTTCCCATGTGCCCTAAG |
| HIF1A_p4 | AATTTAGCGCCAAGTGGAGA | TTTGGCAACTTTGCAAGCTA |
| HIF1A_p5 | GCCATTCATCCGTTCAGAAT | CAATCCAAGGTTGCCAAGTT |
| HIF1A_p6 | TCTTTGAGGGTTGGGATACG | TCAGCCTACTTGGGATTTGG |
| IRF2BP2_p1 | GCAAGAGGGCAGAGTCTACG | ACACACAGGAGCAGGGTTTC |
| IRF2BP2_p2 | GGCGATGAGTTGGTTCATTT | TTCAAGACCTGTCCCTTTGG |
| IRF2BP2_p3 | TTTAAAACAGGCGCACCTTC | ACAGTGGAGGGAGCTGCTTA |
| IRF2BP2_p4 | GGCAGCCAAGGAGAAACA | GTTGACCCAGTCCTGCTCTC |
| IRF2BP2_p5 | CCGTACGCCTGAAGGTTTTA | CGGTGCACTGAAAGAGATCA |
| IRF2BP2_p6 | GACACCCATTTTGTGCAGTG | TCCACTAGCTCCCTGCTGTT |
| LDLR_p1 | CTGGGGAAATCCACTTTTGA | AAATATTTGCTGGCCACAGG |
| LDLR_p2 | TGACCACAGCAGATTCCAAA | GGGAGGCTGAAACATGAGAA |
| LDLR_p3 | GCATTCGGGTTCAAGTGATT | TGGTGAAACCCCGTCTCTAC |
| LDLR_p4 | CCCGAATAGCTGGGATTACA | CACTTTTGGGCAGATCACCT |
| LDLR_p5 | GCTTTGACTGGATGGAGAGC | TACACCCTCCCTCCCTCTCT |
| LDLR_p6 | GCTTTGATGGGTGCTTTGAT | GTGTGGAAGGAAAAGGGACA |
| LSM14A_p1 | TGTTGTGCAACCACCTCATT | GAGCCTGGATCAAAGGTCAA |
| LSM14A_p2 | AGTGGTTTCACGGGATCAAG | AATCATTGCCTTTGCACTCC |
| LSM14A_p3 | ATCCTGGCTCCCTGTAACCT | AAAATTAGCCAAGCGTGGTG |
| NEIL3_p1 | CAGCCATGCAAGTGACTCAT | GGGTCAGGAATTTGGCAATA |
| NEIL3_p2 | TTGCACAGCGGTATTCTCAC | CGCGAATCTTCTCTCCATTC |
| NEIL3_p3 | TAGCCTTTGGTCCTGGAAGA | AAGGGCAATTGGAAGGAGAT |
| NEIL3_p4 | TGGCTTCATTTTCTGGGACT | GCCTAGGAGACATGGGATCA |
| NFAT5_p1 | GGCAGCAAGAGAAAATGAGG | GGACCCAGGAGGTGGTATTT |
| NFAT5_p2 | CATGATGAAACCCTGGCTCT | ATCCTGCCTCAGACTCCAGA |
| NFAT5_p3 | ATCGCCCAAGTCCCTCTACT | TGTCTCCCTGTCTCCCTGTC |
| NFAT5_p4 | AGCACTGGAGCTGATCCCTA | CCGGTGGTTTAAAAAGCGTA |
| NFAT5_p5 | GCCTGACCAATTTGGAGAAA | CCCGAGTAGCTGGGATTACA |
| NIPBL_p1 | GCCAGCGGAATCTACTTGAG | CCACTAGCTACGGCTCCACT |
| NIPBL_p2 | CCTCCCTCCCACTAATCTCC | TATTCCTCCCGTCTGCATTC |
| NIPBL_p3 | TGCCAGCTAAGATTTTGAGGA | AAACCTTTCCAGCCTCAGGT |
| NIPBL_p4 | AATCTACCCCTTCCCCAATG | CCAGAGCCCAAGCATAGAAG |
| NIPBL_p5 | GCTGCCTTGAAAAACAAAGC | CACCAAAGGGATCCAATGAC |
| NIPBL_p6 | TGCACTGTCCTCCGTATTCA | GCAATGTGGCTCAACAGCTA |
| NR3C1_p1 | CTGGCAATGGGTACAAGGTT | CTGTTCCATCACCCTGCTCT |
| NR3C1_p2 | TGGTTCAGAAGCATCCTGTG | CCTGCATGCAAAGAAACTGA |
| NR3C1_p3 | TGGGATCTGCTGAGTCTCCT | AAAGAGTGGGCCTGCAGTAA |
| NR3C1_p4 | GCCCCATTACGCTACACAGT | TCCAGCATTTGAAGCCCTAC |
| NUP153_p1 | AGAGATGGGTCAGCAGCCTA | CTTGAACCATGGAGGTGGAG |
| NUP153_p2 | GGAGGCATGAAGCTAGGATG | CAGTGGCACCTCTGACCATA |
| NUP153_p3 | GGCTCAGACGGTTTTGATTC | GGTTTTGTTGCAGACACACG |
| NUP153_p4 | AAATTGTAGGCCCATGATGC | CCCCCTCCTCAAACACACTA |
| NUP153_p5 | CACCAGTTTTCCCCCAGATA | GTTAAATGGCCAGGCTTGTG |
| NUP153_p6 | TCCTGGTGAGAGGAAACCAC | GGAGGATTAAAGGCGGAGTC |
| OSMR_p1 | GAGGAGGAAGGGAATTGGAG | GCTACTTCTTGCGGGTTCTG |
| OSMR_p2 | AGCACAGGTGGAGAGAGGAA | CAAGTGGCTGGAGAAGAAGG |
| OSMR_p3 | AACTGAGGCCCAAAGAGGTT | TTTTAAAGCAGCCCAGGAGA |
| OSMR_p4 | GAGAAATCCCCTCCTCCAAG | CAAATGCAGAGCGACCAGTA |
| PHF20_p1 | AACGTGACAATTCTGCGTTG | GCTCCACAATGAAACCCTGT |
| PHF20_p2 | TGCACACAGGGTTTCATTGT | ATGCTAGCCAGAGAGCTCCA |
| PHF20_p3 | GCTCTTTCTTGGTGGAGCTG | AAGATGGGCACCAAACACTC |
| PHF20_p4 | CTTGCGCTTTGGGAATATGT | TCCCATATTTGGGGTCTTGA |
| PHF20_p5 | GGGCACACCTTTTTGTGAGT | AAGAGGAAATGGGTGCACTG |
| PHF20_p6 | TTCTGATTTGCAGGTGATGC | AGGCTAACCACCACCTTCCT |
| RICTOR_p1 | GGGAGGAGGAAAGATTGGTC | GTTGGCCTGAAGGACGATAA |
| RICTOR_p2 | GACCCGCAGCATTGATAACT | AACAAACCCATCTCCACTGC |
| RICTOR_p3 | CCCGAGTAGCTGGGACTACA | GATCGAGACCATCCTGGCTA |
| RICTOR_p4 | TGCCTACTCCTGCTTCACCT | CACGCCCACTTACCAATTTT |
| RNF145_p1 | CCAGGAACTATGCCACTGGT | GTCAGATCATGCAGGCCTTT |
| RNF145_p2 | GACGGAGTCTCGCTCTTTTG | GTGGCTGAGGCAGAAGAATC |
| RNF145_p3 | AATGGGCAGATGAAGCAGAC | ACTGGGCCTCAGTATGTTGG |
| RNF145_p4 | CAGGCTCCTTCCTCTTCTCC | AAGAGGGGATGTGGACTCCT |
| RNF145_p5 | ATTCCTGAGATGCCCTTCCT | GAGGCAAAGTTGGAAATGGA |
| RNF145_p6 | GCTAGCAAGGAGTGGGAGTG | GGATGGTAGGCCTCACTGAA |
| ROCK1_p1 | TCCTGAGGACAGCACAGAGA | TCCAAAAGAAGGCAGAAAGG |
| ROCK1_p2 | TCTCACTCTCGCTCTTTCTGG | ACTGGAGAAAGGCGTGTGAC |
| ROCK1_p3 | GTGTGCCTATGGTCCCAGTT | GAGCAGTGGCACAATCTCAA |
| ROCK1_p4 | GCTTTCGCCTTTCCTCTTCT | GCGAGGTGCTTCAGTCTAGC |
| ROCK1_p5 | CGGAAGTGAATTCGGATTGT | TGCAGCGAACCAGACTAATG |
| ROCK1_p6 | CCCGTCAATCTGAACTTGG | AGGCAGGAGAATCACTCGAA |
| SLC38A1_p1 | CATCGTCAGAGAGCTGCAAG | TAACCAAAGCTGCGTGTCAG |
| SLC38A1_p2 | CTGATGCCACTTTTCCACCT | CACCTTCCATCACCTGGAGT |
| SLC38A1_p3 | CCAGGAGGTATTTGGCAATG | GATCCTGGACACACCCTCTC |
| SOS2_p1 | TGCCAGGTGGGAAGTATAGC | GACCTACTGCATTCGTGCAA |
| SOS2_p2 | CTTTCCACGCCTCATCTCTC | TTTGTGCTCTACGTGGCTTG |
| SOS2_p3 | CTTCTAACGGGGAGCAGGTC | AATGCAACAGGCAATTGTGA |
| SOS2_p4 | CTGTAGATGGTGGCGGTCTT | GACAGCTGATCACGCACCTA |
| SOS2_p5 | TCAATTGGTGTTGCCATTGT | CTTAGGATCCCGCATCATGT |
| TGM2_p1 | CTGGTCACTGGGACTGGAAT | CCACAGAACTATGGCCACCT |
| TGM2_p2 | GCAGGTGTGTCTGCATGTCT | CAGATGAGCAGGTGTCCAGA |
| TGM2_p3 | ATCAGGTGAGGCAATGGAAC | CTCCAGGCACACATCAGAGA |
| TGM2_p4 | TCTGGCCACTGAGGTCTCTT | ACCTGGGGGCAGATAGTTCT |
| TRPM7_p1 | TGTAGGCCCAACTGAAATCC | AGGTCCAGCAGAACCAAGAC |
| TRPM7_p2 | CAAACCTTGGCATGTGTGAC | CGCATACACCACACCTTCTG |
| TRPM7_p3 | CAGGCCTAGTTAGCGGAATG | GGGTACCTAGGCGGGACTTA |
| TRPM7_p4 | CTGGCAGATAAGGGAACCAA | TCCAAACCCGTGTCCTCTAC |
| TRPM7_p5 | TGGCAGGCTCGTCTCTTAAT | TGAAACTGGCCAATGGGTAT |
| TRPM7_p6 | TTGGTGTGTGGAGGTAAGCA | GAAGGCACAGTTTGCAGTGA |
