# Supplementary material for: C/EBPβ enhances platinum resistance of ovarian cancer cells by reprogramming H3K79 methylation
Source: Nat Commun. 2018 Apr 30;9:1739. doi: 10.1038/s41467-018-03590-5 (PMC5928165; doi:10.1038/s41467-018-03590-5)
Supplement: Supplementary file 14 — Supplementary Data 11(DOC 77 kb) [file 41467_2018_3590_MOESM14_ESM.doc]

**Supplementary Data 11**. Primers for RT-qPCR

| Gene | Sense primers | Antisense primers |
| --- | --- | --- |
| ABCC1 | TTACTCATTCAGCTCGTCTTGTC | CAGGGATTAGGGTCGTGGAT |
| ABCC3 | ATTCCACTCAACGGAGCTGTG | GCGCGAGTCCTTCAATTTCAT |
| ACACA | TCACACCTGAAGACCTTAAAGCC | AGCCCACACTGCTTGTACTG |
| AMOTL1 | CTGGAACAGTATTGCAGCGG | AGTGAGGTTTTCCGTGGAAGA |
| ATF1 | AGGACTCATCCGACAGCATAG | TTCTGCCCCGTGTATCTTCAG |
| CASC5 | CTTCACACCGAGGACTCAAGA | TTTGATGTGTAGAAGAGGCACTG |
| CDC42BPA | AGACAGCATGTTTTCGTGAAGA | AGCATAGTGCAAGGTTGTAATCC |
| CEBPB | CTTCAGCCCGTACCTGGAG | GGAGAGGAAGTCGTGGTGC |
| DCBLD2 | ATGTGGACACACTGTACTAGGC | CTGTTGGGATAGGTCTGTGGG |
| E2F1 | AGATGGTTATGGTGATCAAAGCC | ATCTGAAAGTTCTCCGAAGAGTCC |
| EGFR | AGGCACGAGTAACAAGCTCAC | ATGAGGACATAACCAGCCACC |
| EREG | GCTCTGCCTGGGTTTCCATC | CCACACGTGGATTGTCTTCTGTC |
| ETS1 | AAACTTGCTACCATCCCGTACGT | ATGGTGAGAGTCGGCTTGAGAT |
| FAT1 | CATCCTGTCAAGATGGGTGTTT | TCCGAGAATGTACTCTTCAGCTT |
| FEV | CACGGCGAGTTCAAGCTCA | CTGGAAGTCGAAGCGGTAGG |
| FOXJ3 | GGAGAGCAGCCTAACGTCTAT | TGGCATAACTGTATGGAGGTTTC |
| FOXL1 | GCCTCGCCCATGCTGTATC | CGTTGAGCGTGACCCTCTG |
| FOXO3 | TCACGCACCAATTCTAACGC | CACGGCTTGCTTACTGAAGG |
| G2E3 | AGAGAGTCCTTACCATGCACC | ACACTCCCGCATTTATTGCTTG |
| GAPDH | GACAGTCAGCCGCATCTTCT | TTAAAAGCAGCCCTGGTGAC |
| GATA1 | CTGTCCCCAATAGTGCTTATGG | GAATAGGCTGCTGAATTGAGGG |
| GATA4 | CGACACCCCAATCTCGATATG | GTTGCACAGATAGTGACCCGT |
| GCM1 | CCAAATCCAGCGGGTAATCTT | GGTGAATGGTATGCAGGAGAC |
| GFOD1 | GACTGACCACATCAAGGGCAT | CGGGCACGTTGAAGTTGAG |
| HIF1A | ATCCATGTGACCATGAGGAAATG | TCGGCTAGTTAGGGTACACTTC |
| HOXA6 | ACCTGCACTTTTCTCCCGAG | CCAAGGGTAAACCGGGCTC |
| INSM1 | GTCCACGCCCGTTTCCTAC | CCAGGTTGAAGCTGCGTTC |
| IRF2BP2 | ACACCCATTTTGTGCAGTGC | ACTGGGACAATAGACCTCTCC |
| IRF3 | AGAGGCTCGTGATGGTCAAG | AGGTCCACAGTATTCTCCAGG |
| IRF4 | ACCCGCAGATGTCCATGAG | GTGGCATCATGTAGTTGTGAACCT |
| KLF4 | ACCAGGCACTACCGTAAACACA | GGTCCGACCTGGAAAATGCT |
| LDLR | AACTGCCATTGTCGTCTTTA | ACATACCCATCAACGACAAG |
| LSM14A | ACCGACAGATCGTCCAATACC | GGACTGAACAATAGCTGGGTCTT |
| MEF2A | GGTCTGCCACCTCAGAACTTT | CCCTGGGTTAGTGTAGGACAA |
| MZF1 | TCCAGGTAGTGTAAGCCCTCA | TCCTGTTCACTCCTCAGATCG |
| NEIL3 | TCTCCTGTTTTGGAAGTGCAG | CATTAGCACATCACCTAGCATCC |
| NFAT5 | GTGGGCAACGACTCTGGAC | GCTTCGACATCAGCATTCCTCA |
| NIPBL | ATGTCCCCATTACTACTCTTGCG | ACCTCTTCTGCTATTCGTGCAT |
| NKX3-1 | CCCACACTCAGGTGATCGAG | GAGCTGCTTTCGCTTAGTCTT |
| NR3C1 | ATAGCTCTGTTCCAGACTCAACT | TCCTGAAACCTGGTATTGCCT |
| NR4A1 | CCCTGAAGTTGTTCCCCTCAC | GCCCTCAAGGTGTGGAGAAG |
| NUP153 | GGACCATCTGGTATATGCCGA | GGCTCCGATGAAGAGAAGGC |
| OSMR | ATGGCTCTATTTGCAGTCTTTCA | CACCCAGATGACATTGGATGTT |
| PHF20 | TAGCTCCTACTGCTGTGGATT | AAGCCGAGGACGTTTTAATGG |
| RICTOR | AGAAGCACGATTTCTAGCCAGT | AGTAGACCTCGCCTTATTTCCA |
| RNF145 | AGTGAACTGGAGTTTGCCTATG | ACACACCACCAACTGACCTATT |
| ROCK1 | AACATGCTGCTGGATAAATCTGG | TGTATCACATCGTACCATGCCT |
| SIX1 | CACCAGTTCTCGCCTCACA | CACCCGATATTTGCCCAC |
| SIX3 | CTGCCCACCCTCAACTTCTC | GCAGGATCGACTCGTGTTTGT |
| SLC38A1 | AACCTCCTTAGGCATGTCTGT | GCAAAGGCGAGTCCCAAAAT |
| SOS2 | ATGTAGAGGAGCGAGTTCAGAA | ATGGTAGTCCACTTTGTACCCT |
| SOX14 | CCACCCTGGGCTACCAGAA | GCCAGTCTTGGTCATGCCT |
| SOX17 | GTGGACCGCACGGAATTTG | GGAGATTCACACCGGAGTCA |
| SOX4 | AGCGACAAGATCCCTTTCATTC | CGTTGCCGGACTTCACCTT |
| SP100 | TCCATGACAAATTGCCTCTCC | GAGATGGGGAACCCGAAGG |
| SPDEF | AGTGCTCAAGGACATCGAGACG | AGCCACTTCTGCACATTGCTG |
| SPI1 | GGGGTGGAAGTCCCAGTAAT | ACGGATCTATACCAACGCCA |
| STAT1 | CCATCCTTTGGTACAACATGC | TGCACATGGTGGAGTCAGG |
| TCF3 | CCGACTCCTACAGTGGGCTA | CGCTGACGTGTTCTCCTCG |
| TCF7L2 | AGAAACGAATCAAAACAGCTCCT | CGGGATTTGTCTCGGAAACTT |
| TEAD4 | GAACGGGGACCCTCCAATG | GCGAGCATACTCTGTCTCAAC |
| TGIF2 | TGACCCCTGGTAGCACACTTA | GTGGTGGCGTGTTGAAGAGT |
| TGM2 | CAAGGCCCGTTTTCCACTAAG | GAGGCGATACAGGCCGATG |
| TRPM7 | ACTGGAGGAGTAAACACAGGT | TGGAGCTATTCCGATAGTGCAA |
| YY1 | CCTCTCAGATCCCAAACAACTG | GCCTTTATGAGGGCAAGCTATT |
| ZBTB7B | GTCCCCAGAGCTACGAACC | AGCTTAGGTAGGCCATCAGGT |
